# Rapid Development of an Integrated Network Infrastructure to Conduct Phase 3 COVID-19 Vaccine Trials

**DOI:** 10.1001/jamanetworkopen.2022.51974

**Published:** 2023-01-03

**Authors:** Alfredo J. Mena Lora, Jessica E. Long, Yunda Huang, Lindsey R. Baden, Hana M. El Sahly, Dean Follmann, Paul Goepfert, Glenda Gray, Beatriz Grinsztejn, Karen Kotloff, Nadine Rouphael, Magdelena Sobieszczyk, Stephen R. Walsh, Jessica Andriesen, Karan A. Shah, Yuanyuan Zhang, Peter Gilbert, Holly Janes, Cynthia L. Gay, Ann R. Falsey, Rebecca L. Tripp, Richard L. Gorman, Tina Tong, Mary Marovich, Kathleen Neuzil, Lawrence Corey, James G. Kublin, COVID-19 Prevention Network

**Affiliations:** Division of Infectious Diseases, Department of Medicine, University of Illinois at Chicago; Department of Medicine, University of Washington, Seattle; Vaccine and Infectious Disease Division, Fred Hutchinson Cancer Center, Seattle, Washington; Statistical Center for HIV/AIDS Research & Prevention, Fred Hutchinson Cancer Center, Seattle, Washington; Division of Infectious Diseases, Brigham and Women’s Hospital, Boston, Massachusetts; Infectious Diseases Section, Department of Medicine, Baylor College of Medicine, Houston, Texas; Department of Molecular Virology and Microbiology, Baylor College of Medicine, Houston, Texas; Biostatistics Research Branch, National Institute of Allergy and Infectious Diseases, National Institutes of Health, Bethesda, Maryland; Division of Infectious Diseases, Department of Medicine, University of Alabama at Birmingham; Perinatal HIV Research Unit, Faculty of Health Sciences, University of the Witwatersrand, Johannesburg, South Africa; South African Medical Research Council, Cape Town, South Africa; HIV/STI Clinical Research Laboratory, Evandro Chagas National Institute of Infectious Diseases–Fundação Oswaldo Cruz, Rio de Janeiro, Brazil; Division of Infectious Disease and Tropical Pediatrics, Department of Pediatrics, and the Center for Vaccine Development, University of Maryland School of Medicine, Baltimore; Hope Clinic of the Emory Vaccine Center, Department of Medicine, Division of Infectious Diseases, Emory University School of Medicine, Decatur, Georgia; Division of Infectious Diseases, Department of Medicine, Columbia University Irving Medical Center, New York, New York; Division of Infectious Diseases, Brigham and Women’s Hospital, Boston, Massachusetts; Vaccine and Infectious Disease Division, Fred Hutchinson Cancer Center, Seattle, Washington; Statistical Center for HIV/AIDS Research & Prevention, Fred Hutchinson Cancer Center, Seattle, Washington; Statistical Center for HIV/AIDS Research & Prevention, Fred Hutchinson Cancer Center, Seattle, Washington; Department of Biostatistics, University of Washington, Seattle; Vaccine and Infectious Disease Division, Fred Hutchinson Cancer Center, Seattle, Washington; Bioinformatics and Epidemiology Program, Fred Hutchinson Cancer Research Center, Seattle, Washington; Division of Infectious Diseases, Department of Medicine, UNC HIV Cure Center, University of North Carolina at Chapel Hill School of Medicine; Infectious Disease Division, Department of Medicine, University of Rochester, Rochester, New York; Vaccine and Infectious Disease Division, Fred Hutchinson Cancer Center, Seattle, Washington; Division of Clinical Development, Biomedical Advanced Research & Development Authority, Washington, DC; Vaccine Research Program, Division of AIDS, National Institute of Allergy and Infectious Disease, National Institutes of Health, Bethesda, Maryland; Vaccine Research Program, Division of AIDS, National Institute of Allergy and Infectious Disease, National Institutes of Health, Bethesda, Maryland; Department of Medicine, University of Maryland, Baltimore; Vaccine and Infectious Disease Division, Fred Hutchinson Cancer Center, Seattle, Washington; Department of Laboratory Medicine and Pathology, University of Washington, Seattle; Vaccine and Infectious Disease Division, Fred Hutchinson Cancer Center, Seattle, Washington; Department of Global Health, University of Washington, Seattle

## Abstract

**IMPORTANCE:**

The COVID-19 pandemic has caused millions of infections and deaths and resulted in unprecedented international public health social and economic crises. As SARS-CoV-2 spread across the globe and its impact became evident, the development of safe and effective vaccines became a priority. Outlining the processes used to establish and support the conduct of the phase 3 randomized clinical trials that led to the rapid emergency use authorization and approval of several COVID-19 vaccines is of major significance for current and future pandemic response efforts.

**OBSERVATIONS:**

To support the rapid development of vaccines for the US population and the rest of the world, the National Institute of Allergy and Infectious Diseases established the COVID-19 Prevention Network (CoVPN) to assist in the coordination and implementation of phase 3 efficacy trials for COVID-19 vaccine candidates and monoclonal antibodies. By bringing together multiple networks, CoVPN was able to draw on existing clinical and laboratory infrastructure, community partnerships, and research expertise to quickly pivot clinical trial sites to conduct COVID-19 vaccine trials as soon as the investigational products were ready for phase 3 testing. The mission of CoVPN was to operationalize phase 3 vaccine trials using harmonized protocols, laboratory assays, and a single data and safety monitoring board to oversee the various studies. These trials, while staggered in time of initiation, overlapped in time and course of conduct and ultimately led to the successful completion of multiple studies and US Food and Drug Administration–licensed or –authorized vaccines, the first of which was available to the public less than 1 year from the discovery of the virus.

**CONCLUSIONS AND RELEVANCE:**

This Special Communication describes the design, geographic distribution, and underlying principles of conduct of these efficacy trials and summarizes data from 136 382 prospectively followed-up participants, including more than 2500 with documented COVID-19. These successful efforts can be replicated for other important research initiatives and point to the importance of investments in clinical trial infrastructure integral to pandemic preparedness.

## Introduction

The COVID-19 pandemic has required an unprecedented global response. A coordinated effort to support safety and efficacy assessments and oversee the development, testing, and clinical evaluation of COVID-19 vaccines was crucial. Critically, to accelerate potential vaccine candidates through testing and approval, public-private partnerships were required that brought government, academic, and private entities together for a coordinated research strategy. In response to these needs, the National Institute of Allergy and Infectious Diseases (NIAID) established the COVID-19 Prevention Network (CoVPN) to assist in the coordination and implementation of phase 3 efficacy trials for COVID-19 vaccine candidates.^1–4^

The purpose of this report is to describe the CoVPN and its contribution to COVID-19 vaccine efficacy trials. This includes some of the key harmonization specifics of trial design, the geographic reach of the studies, the association of study timing with viral variant circulation, and opportunities for cross-protocol analyses and for researchers to access data.

## Inception of the CoVPN

The CoVPN was conceived in April 2020 and became a functional unit within Operation Warp Speed (OWS) at its official designation in May 2020. Operation Warp Speed was a public-private partnership by the US government to facilitate the development, manufacturing, and distribution of COVID-19 vaccines and therapeutics for the US population. This interagency program provided coordination and resource allocation between government agencies and the private sector. The mission of the CoVPN was to design and rapidly operationalize phase 3 clinical trials of multiple vaccine candidates using harmonized protocols (eMethods in Supplement 1) across the various vaccine prototypes in the US government portfolio.^4–8^ The CoVPN aimed to develop an integrated system of clinical trial sites and standardized laboratories that would allow cogent evaluation of each vaccine prototype in the US government portfolio. Comparability between individual vaccines was desired, as it was hoped that multiple vaccines would be effective, and it was clear that different manufacturing times would require different start times between trials. Advancing multiple effective vaccine platforms was needed to mitigate the risk associated with lack of safety and efficacy for some platforms and to diversify the options to meet the global demands.

Aligning with the OWS model, the key to the rapid scale-up was through leveraging existing resources and then forming the CoVPN through a partnership of multiple existing NIAID-funded clinical trial networks, including the HIV Vaccine Trials Network, the HIV Prevention Trials Network, the AIDS Clinical Trials Group, and the Infectious Diseases Clinical Research Consortium.^9^ The broad reach of the CoVPN allowed for inclusion of research sites across the US, South America, and Sub-Saharan Africa (eFigure in Supplement 1). Within the US, the CoVPN sites included NIAID-funded network sites and Veterans Affairs, Native Nations, and the Department of Defense sites.

The multibillion dollar investment from OWS shifted some of the financial risk of vaccine development from pharmaceutical companies to the US government. Phase 3 trials were supported by US government funding through the Biomedical Advanced Research and Development Authority and the NIAID. This model accelerated vaccine development by allowing phases to occur in parallel rather than in sequence; for example, process development and vaccine manufacturing optimization and scale-up could occur while clinical trials were ongoing in anticipation of meeting immunogenicity targets.^10^ As part of the public-private partnership, the clinical trial protocols used in phase 3 trials were overseen by pharmaceutical companies in partnership with US government.^4^ The companies operated as the sponsor of the studies and oversaw operations using contract research organization partnerships. With contract research organization and CoVPN sites together, between 86 and 130 sites enrolled in each trial (eTable 4 in Supplement 1).

Extensive discussion during protocol development occurred through the Accelerating COVID-19 Therapeutic Interventions and Vaccines Working Group, a forum run under the administrative auspices of the Foundation for the NIH that brought together academic, government regulatory, and industry executives to discuss both issues of trial design and implementation. This group facilitated the harmonized end point discussion of statistical methods and was critical for communication with companies in an open and transparent fashion. European and US regulators were frequent attendees. These discussions were instrumental in allowing study enrollment, clinical end points, and methods for follow-up to be harmonized across studies, allowing for pooling of resources in outreach and enrollment and the use of unified procedures.^3^ As a result, data emerging from these studies could be analyzed across vaccine candidates, providing a unique database to assess correlates of risk and protection across all OWS-supported studies.

## Vaccine Platforms

All the vaccines in the US government programs used the SARS-CoV-2 spike protein as the immunogen; 4 of the 5 utilized the 2/SP version^11^ of the prefusion structure and 1 vaccine (AstraZeneca/Oxford) utilized the full-length spike protein. The design of the program was based on the premise that a diversity of platforms would be evaluated to diversify the manufacturing supply capacity and as potential differences in safety and efficacy were likely to be seen. It was recognized that SARS-CoV-2 was a global problem and that vaccination of the entire global population would be required. As such, multiple platforms were purposely selected for within the pipeline (eTable 1 in Supplement 1). Each of the vaccine candidates were tested in phases 1 and 2 clinical trials to define the maximal tolerated dose in adults aged 18 to 55 years and then 55 years and older. Binding and neutralizing antibody responses were assessed relative to panels of convalescent plasma from patients recovering from COVID-19. All vaccine candidates demonstrated immunogenicity and an acceptable safety profile prior to phase 3 testing.

## Phase 3 Study Designs

### Harmonization of End Points and Statistical Analysis Plan

The designs of the 5 phase 3 studies were similar in relative size and the number of end points to time to analysis. The studies were modeled in collaboration with the CoVPN statistical team led by Peter Gilbert with the World Health Organization Solidarity Vaccine Trial Expert Group, the US Food and Drug Administration (FDA) led by Marion Gruber, PhD, MS, and Peter Marks, MD, PhD,^12,13^ and the NIAID Data Safety Monitoring Board (DSMB)^12^ (Table 1). Large sample sizes enabled rapid collection of end points, with large safety data for public reassurance. At the study design level, primary end points were selected to reflect 2 key priorities: identifying vaccine efficacy against symptomatic COVID-19 disease as well as against various levels of disease severity.^3^ Vaccine efficacy against symptomatic disease is crucial as it has correlated with efficacy against severe disease in other respiratory viral diseases, which is the main driver of hospitalization. Primary study end points, while differing slightly across studies, were harmonized to use the concordant definitions of mild, moderate, severe, and critical COVID-19 disease based on criteria from the Centers for Disease Control and Prevention (CDC). A statistical analysis plan (SAP)^15^ was developed by the CoVPN statistical team in collaboration with the COVID-19 Response Biostatistics Team.

### End Point Definitions

The primary analyses of all trials evaluated vaccine efficacy based on results of serology and polymerase chain reaction testing. For all studies, the clinical case ascertainment of COVID-19 was based on the CDC criteria with minor differences in definitions of primary end points. All studies required a positive molecular test result and usually 2 or more COVID-19 symptoms. In most studies, participants used mobile technologies to submit electronic diaries that enabled constant communication with the study team. This led to early identification of COVID-19–like symptoms and access to early molecular testing for case detection. The statistical analyses and interim monitoring of the vaccine efficacy against the primary clinical end points have also been harmonized, facilitated by the CoVPN statistical team and a common DSMB across all 5 trials.

All the studies were designed to accommodate a 50% to 60% vaccine efficacy with a 30% lower bound, requiring approximately 150 primary end points per study. It was estimated this would occur about 6 months after onset of enrollment, though it was recognized that this would vary by symptomatic infection rates.

### Differences in Design Across Studies

The phase 3 study designs had minor platform-specific differences, such as the primary efficacy assessment window and the vaccine dose regimen (Table 1). The COVE (Coronavirus Vaccine Efficacy and Safety) study (Moderna)^16^ and AZD1222 (AstraZeneca)^17^ evaluated 2 doses 28 days apart with the window of efficacy opening at 14 days after the second dose for both trials. The ENSEMBLE study (Janssen/Johnson & Johnson)^18,19^ assessed a single dose of Ad26.COV2.S with the efficacy measured 14 days after the initial dose. The PREVENT-19 (Prefusion Protein Subunit Vaccine Efficacy Novavax TRIAL COVID-19) study (Novavax)^20^ had a dosing interval of 2 doses of NVX-CoV2373 21 days apart, measuring efficacy from 7 days after the second dose. The VAT0008 study (Sanofi Pasteur)^21^ included 2 doses, 21 days apart, with efficacy measured beginning 14 days after the second dose. Minor differences in inclusion and exclusion criteria, secondary efficacy end points, and safety end points across the studies are detailed in Table 1. The first 4 studies were designed to follow participants for 2 years post injection, while VAT0008 was designed with 1 year of follow up.

### Single DSMB

A single DSMB with 11 members was established for review and monitoring of all CoVPN vaccine studies.^22^ This approach leveraged the expertise and insights gained from involvement in the entire CoVPN clinical end points that were met.^23^ With access to unblinded interim data, the single DSMB responded to real-time data to address safety and efficacy concerns and guide changes introduced by amendments to the study protocols.

### Casting a Wide Net: Sites and Demographics

The pandemic resulted in higher morbidity and mortality in elderly populations and a disproportionate impact in racial and ethnic minority communities.^24^ The CoVPN sites engaged in extensive community outreach across the US and international sites for these important phase 3 studies.^25,26^ This led to rapid deployment of the trials along with broad demographic representation. The wide geographic distribution of vaccine trial sites addressed significant issues related to equity and representation, including international representation; the 5 phase 3 trials included sites in 16 countries across 5 continents. The diversity in the US-based COVE trial^16^ was high, with racial and ethnic minority groups accounting for 56% of enrollment. Diligence and conscious monitoring of the trial’s demographic composition ensured that the racial and ethnic diversity in the trials represented the diversity of the US outbreak. Outreach was also specifically targeted to elderly populations who are at greater risk of severe COVID-19 disease; as a result, nearly one-quarter of study participants in COVE were 65 years or older. Additional details of each protocol’s demographic and clinical characteristics are in eTable 2 in Supplement 1.

The harmonization of outreach and equitable enrollment efforts were aided by a screening registry hosted on a CoVPN website, which allowed potential participants to register to be recruited as study participants at CoVPN study sites across the US. Importantly, the CoVPN clinical research sites have decades of experience with deep and meaningful community engagement. Efforts to enroll racial and ethnic minority groups included extensive community outreach with trusted community leaders and in community anchors such as barber shops and churches. Challenges with minority enrollment were evident prior to the COVID-19 pandemic. A review of HIV vaccine studies in the US^24^ showed a 94% increase in enrollment of racial and ethnic minority individuals, from 17% to 32.7%, between 1988 and 2002. Partnerships and community engagement contributed to this increase in recruitment and retention in minority communities.^27^ This experience was leveraged for COVID-19 vaccines and led to the diverse enrollment seen in the CoVPN-affiliated studies.^28^ This success was a result of considerable and focused effort and reflects ongoing difficulties in recruitment and retainment in these communities. At CoVPN sites, enrollment of White participants outpaced enrollment of racial and ethnic minority participants, so achieving enrollment goals required implementation of strategies such as setting predetermined targets or pausing enrollment of White non-Hispanic participants to increase diversity throughout the study. These strategies were also used to ensure enrollment of older populations at high risk for severe COVID-19 disease. Without these targeted CoVPN efforts, the level of racial, ethnic, and age diversity seen in the phase 3 studies would not have been reached.

Overall, data from the COVE, PREVENT-19, ENSEMBLE, and AZ1222 trials showed that 47% of participants enrolled at CoVPN sites in the US were racial and ethnic minority individuals.^29^ Inclusion of racial and minority groups is of great importance given the disproportionate impact of the COVID-19 pandemic on their communities and historical issues of access and representation in clinical studies. In our subsequent CoVPN-designed harmonized immune correlates studies, 50% of US participants selected for immunogenicity measurements were underrepresented racial and ethnic minority individuals. The overall demographics of each clinical trial demonstrate the global effort of the CoVPN (eTable 2 in Supplement 1), and study site performance also illustrates the contributions of both public and private institutions to the overall effort (eTable 4 in Supplement 1).

## Timelines and Milestones

### Trial Onset and Effect of Variants on Study Timelines

**Figure 1.**
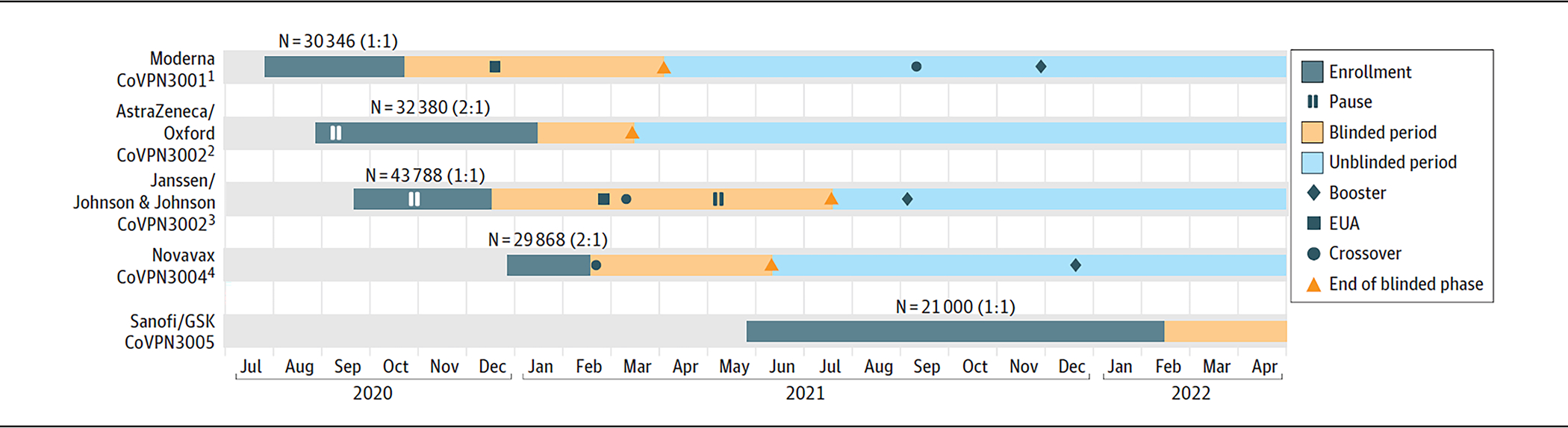
COVID-19 Prevention Network Studies 3001 to 3005 Analysis-Ready Blinded Phase Data Summary as of November 15, 2021 EUA indicates emergency use authorization.

The time for manufacturing, initiation of the phase 1 trials, and the transition to each phase 1 or 2 trial differed by platform, resulting in staggered start dates for the 5 phase 3 trials (Figure 1 and eTable 3 in Supplement 1). Each of the trials was designed to rapidly enroll, evaluate durability, and continue follow-up for a 1- to 2-year period.

**Figure 2.**
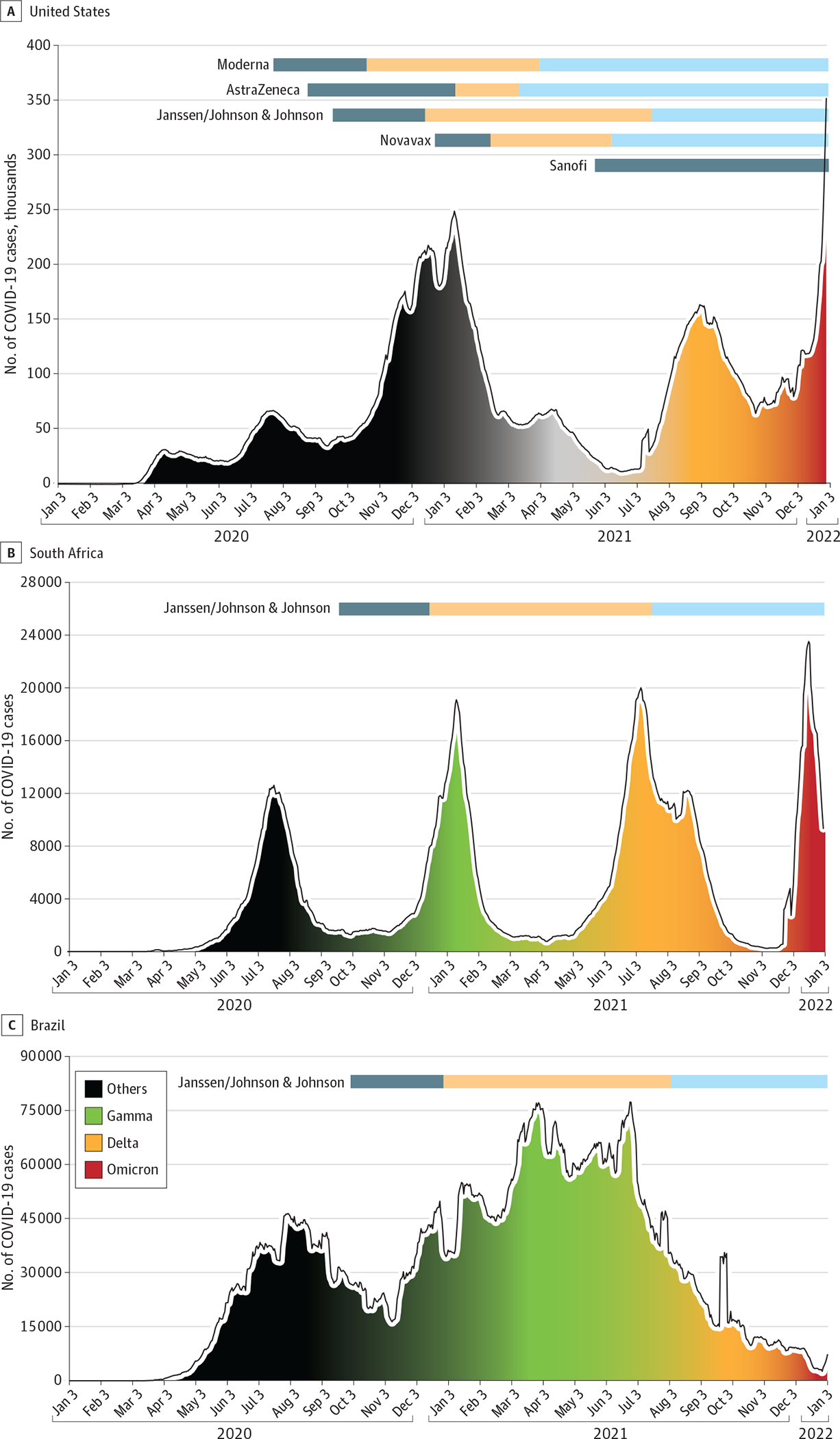
Predominant Circulating COVID-19 Variant Timeline Overlaid With Vaccine Clinical Trials in the US, South Africa, and Brazil

Staggered trial onset and varied geographic distribution introduced several challenges in comparability of vaccine platforms, including heterogeneity of public health mitigating measures, the impact of circulating SARS-CoV-2 variants, and local emergency authorization of COVID-19 vaccines. Circulating variants of concern emerged in each study dependent on timing and geography. Figure 2 demonstrates the timing of study launch in the 3 countries with the highest number of clinical trial sites: the US, South Africa, and Brazil. The varied launch dates of the phase 3 trials resulted in different vaccines being tested against different dominant viral strains in each country (Figure 2). The COVE study, the CoVPN’s first phase 3 study, enrolled at a time when no major variants of concern were reported in the US. Multinational trials had wider exposure to variants of concern (Figure 2B and C).

### Crossover Designs and Participant Unblinding

In the US, due to the availability of COVID-19 vaccines under emergency use authorization (EUA), participants enrolled in the COVE, ENSEMBLE, and AZD1222 trials became eligible for unblinding after December 14, 2020, allowing placebo recipients to receive active vaccines available through their study or EUA eligibility. Approaches to respond to the availability of EUA vaccines differed by study, with some using crossover designs (eTable 3 in Supplement 1). Participants in the COVE study were unblinded and those who received placebo were offered open-label messenger RNA (mRNA)–1273 vaccine starting shortly after EUA in December 2020, concomitant with a redesign of the statistical approach to allow continued learning from these vanguard volunteers.^16^ Participants in the AZD1222 study, which has not received an EUA in the US, were unblinded ahead of schedule to allow placebo recipients to receive an alternative vaccine available through EUA. For the ENSEMBLE study, the open-label phase started on February 22, 2021, following EUA of this vaccine. Participants in the PREVENT-19 study, which began enrollment shortly after other vaccines became available through EUA, could opt to unblind after receiving the study injection if they wished to pursue an EUA vaccine. Beginning on April 20, 2021, PREVENT-19 launched a blinded crossover study phase, in which all blinded participants received a second set of injections with the opposite assignment from the original injection, resulting in all active participants receiving a vaccine by the end of the crossover phase. The VAT0008 study, which began enrollment after several other vaccines were available, included prior coronavirus vaccination as an exclusion criterion, seeking vaccine-naive populations. As authorized vaccines became available, VAT0008 study participants could opt to be unblinded if they wished to pursue a locally available EUA vaccine.

### Booster Vaccinations

In response to evidence of waning immunity after the Delta variant surge,^25,26^ booster doses of vaccines were incorporated into some study designs (eTable 3 in Supplement 1). The COVE study offered booster doses of mRNA-1273 beginning September 2021. The ENSEMBLE study began offering a second dose in October 2021, following the results of the ENSEMBLE-2 study testing a 2-dose regimen.^30,31^ The AZD1222 study did not provide booster vaccinations but encouraged participants to seek EUA vaccine boosters when they became available. A booster vaccine was provided to adult PREVENT-19 study participants beginning December 20, 2021.

### Study Pauses and Safety Assessments

Two studies inserted brief pauses to investigate safety concerns (eTable 3 in Supplement 1). The AZD1222 trial was placed on hold due to an event of transverse myelitis reported in a different clinical trial investigating the same ChAdOx1-S/nCoV-19 (AstraZeneca) COVID-19 vaccine. The ENSEMBLE study experienced brief study pauses due to a reported unexplained illness and blood clots in the context of a vaccine-inducted thrombotic thrombocytopenia. In response, amendments to the protocol were added to address additional safety measures.

### Study Outcomes and the Impact of Variants

All the OWS-sponsored vaccine candidates had positive phase 3 results (Table 2), underscoring the importance of immune responses directed to the spike protein in protective immunity to SARS CoV-2. In primary analyses, vaccine efficacy was the highest for the 2-dose regimens of mRNA-1273 (Moderna) (94.1%), followed by NVX-CoV2373 (Novavax) (90.4%) and ChAdOx1-S/nCoV-19 (AstraZeneca) (74.0%), was 66.9% for the single-dose regimen of Ad26.COV2.S (Janssen/Johnson & Johnson), and was 64.7% for the 2-dose regimen of the bivalent vaccine with AS03-adjuvant (sanofi/GSK). All studies demonstrated high vaccine efficacy against secondary outcomes as well (Table 3). Variability in the spike protein can affect antibody neutralization and thus vaccine efficacy, especially since the primary end point for all trials centered around mild to moderate COVID-19.^32^ In the final analysis of each of the 3 studies that completed the blinded phase, a reduction in vaccine efficacy was seen in comparison with the primary analysis (Table 3); this reduction was likely due to a combination of waning immunity and the emergence of variants of concern.

### Measuring Vaccine Efficacy After Crossover

Given the context of vaccine availability through EUA and use of crossover designs, the COVID-19 Response Biostatistics Team developed novel statistical approaches that enabled the assessment of vaccine durability and vaccine efficacy against emerging variants after the placebo group was vaccinated with the study product.^33–35^ Critical considerations included the following: (1) traditional unblinding of the placebo group affects our ability to obtain longer-term efficacy and safety data, given the additional confounding factors; (2) an open-label design may lead to changes in behavior by participants and could confound estimates of vaccine efficacy; and (3) a blinded crossover design minimizes these concerns, as this design consists of providing the original vaccine group placebo and providing vaccine to the placebo group, and thus providing timely access to the vaccine for those in the placebo group while allowing the study to remain blinded. Continued follow-up after crossover can double the number of participants who can contribute to postvaccination immune correlate analyses as well as safety assessments. Crossover design also helps studies adapt to the need for additional booster doses. As described herein, of the CoVPN-supported studies, PREVENT-19 was the only one to include a blinded crossover design, while the COVE and ENSEMBLE trials included unblinded crossover activity. In these studies, as well as ADZ1222, participants had the option to unblind and seek approved vaccines outside of the study. Many individuals remained in the studies and were followed up, while others chose to leave the studies altogether. The unblinding process was an important and labor-intensive task that involved significant patient education on all available vaccine options.

### Cross-Platform Analyses: a Bridge Between Studies, a Bridge to the Future

The concept of a cross-platform approach, in which data from multiple trials can be both shared and analyzed, is the final and natural step in the process of harmonization between the trials. The cross-platform concept allows for sharing of data from the studies to help bridge gaps in understanding and leverage the 136 382 participants to better answer key research questions and guide policy decisions.

### Analysis of Immune Correlates

Harmonization of end points and SAPs allowed for the statistical design and analysis of immune correlates that were shared among CoVPN phase 3 trials. The SAP developed by the CoVPN statistical center in collaboration with the COVID-19 Response Biostatistics Team served as a master-protocol SAP for harmonized immune correlates analyses across all CoVPN studies and as a guide for non-CoVPN studies. The SAP was shared with the broader scientific community in May 2020 and subsequently edited. This transparency is crucial during a public health emergency to increase trust and improve collaboration. The immune correlates of the SAP have since been applied to the analysis of the COVE, ENSEMBLE, and PREVENT-19 trials.^19,36^

### Harmonization of Laboratory Data

Laboratory data are key to understanding the impact of vaccines and to analyze end points for immune correlates of protection. All protocols evaluated similar correlates of protection across partner laboratories as per the master protocol SAP. These assays were developed with the assistance of the FDA, often after the trials were initiated. Though studies used different laboratory partners, serologic immune response assays were validated and calibrated to a common scale based on the World Health Organization anti–SARS-CoV-2 immunoglobulin international standard sample^37,38^ to provide the ability to pool and compare results. Comparing correlates of protection across studies that encompass a wide geographic and demographic distribution can provide great insights into vaccine effectiveness and account for different groups, such as elderly or immunocompromised hosts. The multitrial data can also be used to assess the impact after 1 dose or multiple doses, and across a wider spectrum of time with different circulating variants.

### Data and Post Hoc Analysis

Statisticians at CoVPN worked closely with the single DSMB to weave a constant thread across parallel studies, connecting dots and insights from lessons learned between them. This approach should serve as a model for future studies answering public health emergencies. The CoVPN statisticians will have access to data from all studies, and the application of these data to analyses not included in the protocols will be a unique opportunity to answer a multitude of questions that no single study could accomplish. With the harmonization of clinical and laboratory end points, the direction of future investigations outlined hereinafter will be enabled by the data across these studies.

### Future Directions

The rich data obtained from 5 randomized clinical trials will help researchers answer key questions about COVID-19 vaccines and COVID-19 itself. Through these large data sets, cross-platform, post hoc analyses began focusing on specific public health needs and emerging questions. The cross-platform approach can leverage the data of over 136 382 participants, including several thousand with asymptomatic infections and 2539 with documented COVID-19 in varying degrees of severity, to answer questions that would be underpowered and difficult to answer in a single trial. Examples include COVID-19 risk factors and vaccine effectiveness for specific at-risk groups, especially through combining the placebo groups, and to further understand the impact of vaccines on postacute complications of COVID-19.

Understanding safety, immunogenicity, and vaccine efficacy in special populations such as those living with HIV are important questions that pooled multitrial data can help answer. These special populations comprise a minority of recruitment and enrollment in each study and severely immunocompromising conditions were mostly excluded from the trials. However, pooled data of patients living with HIV and other conditions may provide important insights. The effect of variants, both on the populations at large and within these special communities, can also be better understood and studied by combining data from all the trials. Cross-protocol analyses can help identify differences in immunogenicity across vaccine platforms between previously infected participants in the placebo and vaccine arms across trials and assess the effect on COVID-19 incidence in these groups. Pooling these data can help clarify the impact of previous exposure to COVID-19 on vaccine effectiveness.

Cross-platform analysis can be used to define COVID-19 risk factors in vaccinated and unvaccinated participants. Defining risk factors that contribute to decreased vaccine effectiveness for different subgroups, such as differences between age groups and body mass index, can help public health officials guide recommendations. Specific environmental risk factors such as occupation, use of public transportation, and local public health mitigation measures that can impact the force of exposure can be compared among sites and across vaccine platforms to better understand case rates between groups. As nonpharmacological interventions and COVID-19 mitigations recede in many countries, well-defined risk factors can help identify and educate individuals at risk to continue safety measures and seek early treatments to prevent progression when breakthrough infections occur. Resources for researchers to access data are now available,^39^ with key information on available studies, results, and future directions.

## Conclusions

The COVID-19 pandemic caused over 400 million cases and 6 million deaths in just 2 years.^40^ Within weeks of the start of the crisis, the US government leveraged industry, government, and academic resources toward the study of COVID-19 vaccines and monoclonal antibodies in diverse populations worldwide. This effort leveraged the expertise of existing research networks, established a joint DSMB, deployed 5 phase 3 clinical trials with harmonized end points, and ultimately led to the successful completion of multiple studies and FDA-authorized vaccines in less than a year. Large sample sizes were feasible during the pandemic and contributed to the achievement of end points and large safety data. This cross-platform approach led to harmonization of data collection across trials and the ability to analyze data from all studies, a novel approach that will continue to yield answers to pressing questions and help guide public health policy. Importantly, this success can be replicated for other important research initiatives and serve as a standard for future clinical studies. Investment and use of this collaborative cross-platform model will continue to provide answers to pressing COVID-19 questions and serve as a model for future pandemics.

## Supplementary Material

Supplement 2 Nonauthor Collaborators Group Information: A list of the COVID-19 Prevention Network members Nonauthor Collaborators

Supplement 1 eMethods. Trial Protocols and Inception of the CoVPN **eMethods.** Trial Protocols and Inception of the CoVPN

Supplement 1 eFigure. Location of CoVPN Clinical Trial Sites Worldwide and in the US **eFigure.** Location of CoVPN Clinical Trial Sites Worldwide and in the US

Supplement 1 eTable 1. Vaccine Platforms and Phase 3 Vaccine Efficacy Studies **eTable 1.** Vaccine Platforms and Phase 3 Vaccine Efficacy Studies

Supplement 1 eTable 2. Demographic and Clinical Characteristics of Each Study Population at Baseline **eTable 2.** Demographic and Clinical Characteristics of Each Study Population at Baseline

Supplement 1 eTable 3. Clinical Trial Milestones **eTable 3.** Clinical Trial Milestones

Supplement 1 eTable 4. Individual Study Site Enrollments by Phase 3 Clinical Trial

## Figures and Tables

**Table 1.** Clinical Trial Designs

	Clinical trial				
Trial characteristic	COVE (CoVPN3001)	AZD1222(CoVPN3002)	ENSEMBLE (CoVPN3003)	PREVENT-19 (CoVPN3004)	VAT00008(CoVPN3005)
Location of study sites	US	Chile, Peru, and US	Argentina, Brazil, Chile, Colombia, Mexico, Peru, South Africa, and US	Mexico and US	Colombia, Ghana, Honduras, India, Japan, Kenya, Mexico, Nepal, Nigeria, Uganda, Ukraine, and US
Key eligibility criteria^a^	Aged ≥18 y and with locations or circumstances that put them at an appreciable risk of SARS-CoV-2 infection, a high risk of severe COVID-19, or both	Aged ≥18 y, with medically stable conditions, and increased risk for SARS-CoV-2 infection, including high risk for symptomatic and severe COVID-19	Aged ≥18 y, in good or stable health, and no coexisting conditions that have been associated with an increased risk of severe COVID-19	Aged ≥18 y, with substantial risk of SARS-CoV-2 exposure, and medically stable	Aged ≥18 y, not intending to receive approved COVID-19 vaccine
Key exclusion criteria^a^	Known history of SARS-CoV-2 infection	History of laboratory-confirmed SARS-CoV-2 infection; any confirmed or suspected immunosuppressive or immunodeficient state	Clinically significant acute illness	Known previous laboratory-confirmed SARS-CoV-2 infection; known immunosuppression	Clinically significant medical condition and prior administration of COVID-19 vaccine
Primary dose regimen	2 Doses, 28 d apart	2 Doses, 28 d apart	1 Dose	2 Doses, 21 d apart	2 Doses, 21 d apart
Primary safety end points	Solicited local and systemic ARs through 7 d after each dose of IP; unsolicited AEs through 28 d after each dose of IP; MAAEs or AEs leading to withdrawal (entire study); SAEs (entire study)	Incidence of AEs for 28 d after each dose of IP; incidence of SAEs, MAAEs, and AESIs from 1 d post treatment through 730 d; incidence of local and systemic solicited AEs for 7 d after each dose of IP	Occurrence and relationship of SAEs (entire study), MAAEs (<6 mo post vaccination), and MAAEs leading to study discontinuation (entire study)	Incidence and severity of MAAEs and unsolicited AEs through 28 d after second injection of IP; incidence and severity of MAAEs attributed to study vaccine, SAEs, and AESIs through 2 mo, and during 12 through 24 mo or the end of study; death due to any cause	Unsolicited injection site and systemic AEs reported in the 30 min after each IP; unsolicited injection site and systemic AEs through 21 d after injection of IP; presence of MAAEs, SAEs, and AESIs throughout the study; reactogenicity subset with solicited AE 7 d after each IP
Primary efficacy end point	First occurrence of PCR-confirmed, mild, moderate, or severe to critical COVID-19, in seronegative participants	First occurrence of PCR-confirmed, mild, moderate, or severe to critical COVID-19, in seronegative participants	First occurrence of PCR-confirmed, moderate to severe to critical COVID-19, in seronegative participants	First occurrence of PCR-confirmed, mild, moderate, or severe to critical COVID-19, in seronegative participants	First occurrence of PCR-confirmed symptomatic COVID-19
Start of primary efficacy end point	14 d After second dose	14 d After second dose	14 d After vaccination	7 d After second dose	14 d After second dose
Follow-up period per participant	2 y	2 y	2 y	2 y	iy
Key secondary efficacy end points^b^	Severe COVID-19 (≥14d after second dose)	Proportion of participants positive for SARS-CoV-2 infection (measured through nucleocapsid antibody seroconversion)	First occurrence of molecularly confirmed, moderate or severe to critical COVID-19, with onset 28 d post vaccination, in seronegative participants	First episode of PCR-positive COVID-19 shown by gene sequencing to represent a variant not considered as a variant of concern or interest according to the CDC's SARS-CoV-2 variant classifications and definitions^14^	Occurrence of SARS-CoV-2 infection and severe COVID-19 illness (≥14 d after second dose); stage 2 results are for the bivalent vaccine only.
Disease definition (all require PCR or NAAT confirmation of SARS-CoV-2 infection in addition to listed symptoms)	Cough, shortness of breath, or pneumonia (≥1); or fever (≥38 °C), chills, myalgia, headache, sore throat, anosmia, or ageusia (≥2)	1 d of Fever, dyspnea, or shortness of breath or ≥2 d of chills, cough, myalgia, fatigue, headache, vomiting, diarrhea, anosmia, ageusia, sore throat, congestion, runny nose	Respiratory symptoms or DVT (≥1); or FDA criteria for moderate COVID-19, anosmia, ageusia, red or bruised-looking feet or toes (≥2)	Fever or cough (≥1); or FDA criteria for mild COVID-19, shortness of breath, anosmia, or ageusia (≥2)	New onset of fever, shortness of breath, altered level of consciousness, myocarditis, thromboembolic event, purpura fulminans, pneumonia, chilblains (≥1) or >24 h of cough, anosmia, ageusia or ageusia (≥1) or >24 h sore throat, chills, myalgia, fatigue, malaise, headache, rhinorrhea, abdominal pain, nausea, diarrhea, or vomiting (≥2)
Severe disease definition^c^	CDC criteria for severe or critical	CDC criteria for severe or critical	CDC criteria for severe or critical	CDC criteria for severe or critical	CDC criteria for severe or critical

Abbreviations: AE, adverse event; AESI, adverse event of special interest; AR, adverse reaction; CDC, Centers for Disease Control and Prevention; DVT, deep vein thrombosis; FDA, US Food and Drug Administration; IP, investigational product; MAAE, medically attended adverse event; NAAT, nucleic acid amplification test; PCR, polymerase chain reaction; SAE, serious adverse event.

a A full list of inclusion and exclusion criteria can be found at the protocol links provided in the eMethods in Supplement 1.

b A full list of secondary end points can be found at the protocol links provided in the eMethods in Supplement 1.

c Described in text.

**Table 2.** Summary of Clinical Trial Results

	Clinical trial									
	COVE		AZD1222		ENSEMBLE		PREVENT-19		VAT00008 (stage 2)^a^	
Treatment group	Placebo	mRNA- 1273	Placebo	AZD1222	Placebo	Ad26.COV2.S	Placebo	NVX-CoV2373	Placebo	CoV2 preS dTM-AS03 (D614+ B.1.351)
Study population, No.										
Full-analysis^b^	15 166	15 180	10 797	21 583	21892	21 896	9961	19 907	6450	6472
Per protocol^c^	14164	14287	8550	17 662	19398	19 400	8140	17312	5680	5736
End points, No. of events										
Primary: virologically confirmed symptomatic COVID-19	744	55	184	141	895	443	63	14	89	32
Secondary: severe COVID-19	106	2	10	1	176	46	4	0	2	3

Abbreviation: mRNA, messenger RNA.

a Results from stage 2 report on the bivalent vaccine data only.

b Indicates cohort of participants who had undergone randomization and received at least 1 dose of placebo or vaccine.

c Indicates cohort of participants who were SARS-CoV-2 negative before the first dose and received the complete number of doses, with no major Protocol deviation.

**Table 3.** Vaccine Efficacy Results of Clinical Trials

	Clinical trial, VE, estimate (95% CI)				
End point^a^	COVE	AZD1222	ENSEMBLE	PREVENT-19	VAT00008 (stage 2)
Primary analysis					
Primary VE	94.1 (89.3–96.8)	74.0 (65.3–80.5)	66.9 (59.0–73.4)	90.4 (82.9–94.6)	64.7 (46.6–77.2)
Secondary VE	100.0 (NE-100.0)	64.3 (56.1–71.0)	66.1 (55.0–74.8)	100.0 (85.8–100.0)	NA
Final blinded phase					
Primary VE	93.2 (91.0–94.8)	67.0 (58.9–73.5)	56.3 (51.3–60.8)	NA	NA
Secondary VE	98.2 (92.8–99.6)	61.0 (54.4–66.7)	52.9 (47.1–58.1)	NA	NA

Abbreviations: NA, not applicable; NE, not estimable; VE, vaccine efficacy.

a Primary and secondary end points differ by study; refer to Table 1 for definition of primary end points. Each study had multiple secondary end points; the results reported here refer to the key secondary end point listed in Table 2.
