# Error in Figure

**DOI:** 10.1001/jamanetworkopen.2023.17317

**Published:** 2023-05-22

In the Special Communication titled “Rapid Development of an Integrated Network Infrastructure to Conduct Phase 3 COVID-19 Vaccine Trials,”^1^ published January 23, 2023, the graphs appearing in panels A and C of Figure 2 were inadvertently transposed. This article has been corrected.
