# Supplementary material for: Rapid Development of an Integrated Network Infrastructure to Conduct Phase 3 COVID-19 Vaccine Trials
Source: JAMA Netw Open. Author manuscript; Available in PMC 2023 Oct 3. (PMC10546713; doi:10.1001/jamanetworkopen.2022.51974)
Supplement: Supplement 2 Nonauthor Collaborators Group Information: A list of the COVID-19 Prevention Network members — Nonauthor Collaborators [file NIHMS1927825-supplement-Supplement_2_Nonauthor_Collaborators_Group_Information__A_list_of_the_COVID-19_Prevention_Network_members.pdf]

Supplemental Online Content: Nonauthor Collaborators
*First name, last name, and suffix (if applicable) are required and will appear in PubMed.

*Group Name(s): COVID-19 Prevention Network							
***First Name and Middle Initial(s)**	***Last Name**	***Suffix (eg, Jr, III)**	Academic Degrees	Institution	Location (city, state/province, country)	Role or Contribution, eg, chair, principal investigator	Group (if more than 1 Group listed in the byline) and/or Subgroup (eg, Steering Committee)
Daniel	Reirden		MD	Anschutz Medical Campus (NIAID/DAIDS name: Children's Hospital Colorado CRS)	Aurora, CO, US	Patient enrollment and data entering	COVID-19 Prevention Network
Lilly	Immergluck		MD	Morehouse School of Medicine	Atlanta, GA, US	Patient enrollment and data entering	COVID-19 Prevention Network
Colleen	Kelley		MD	The Ponce de Leon Center CRS	Atlanta, GA, US	Patient enrollment and data entering	COVID-19 Prevention Network
Anna	Durbin		MD	JHU CIR CRS	Baltimore, MD, US	Patient enrollment and data entering	COVID-19 Prevention Network
Milagritos	Tapia		MD	University of Maryland School of Medicine VTEU	Baltimore, MD, US	Patient enrollment and data entering	COVID-19 Prevention Network
Megan	Deming		MD	University of Maryland School of Medicine VTEU	Baltimore, MD, US	Patient enrollment and data entering	COVID-19 Prevention Network
Karen	Kotloff		MD	University of Maryland School of Medicine VTEU	Baltimore, MD, US	Patient enrollment and data entering	COVID-19 Prevention Network
Jorge	Pinto		MD	SOM Federal University Minas Gerais Brazil NICHD CRS	Belo Horizonte, BR	Patient enrollment and data entering	COVID-19 Prevention Network
Paul	Goepfert		MD	Alabama CRS	Birmingham, AL, US	Patient enrollment and data entering	COVID-19 Prevention Network
Zaheer	Hoosain		MD	Josha Research CRS	Bloemfontein, RSA	Patient enrollment and data entering	COVID-19 Prevention Network
Johan	Lombaard		MD	Josha Research CRS	Bloemfontein, RSA	Patient enrollment and data entering	COVID-19 Prevention Network
Kathryn	Stephenson		MD	Beth Israel Deaconess Medical Center	Boston, MA, US	Patient enrollment and data entering	COVID-19 Prevention Network
Lindsey	Baden		MD	Brigham and Women's Hospital Vaccine CRS	Boston, MA, US	Patient enrollment and data entering	COVID-19 Prevention Network
Stephen	Walsh		MD	Brigham and Women's Hospital Vaccine CRS	Boston, MA, US	Patient enrollment and data entering	COVID-19 Prevention Network
Ken	Mayer		MD	Fenway Health CRS	Boston, MA, US	Patient enrollment and data entering	COVID-19 Prevention Network
Pedro	Cahn		MD	Fundacion Huesped CRS (BA- Amalgro CRS)	Buenos Aires, ARG	Patient enrollment and data entering	COVID-19 Prevention Network

*First name, last name, and suffix (if applicable) are required and will appear in PubMed.

*First Name and Middle Initial(s)	*Last Name	*Suffix (eg, Jr, III)	Academic Degrees	Institution	Location (city, state/province, country)	Role or Contribution, eg, chair, principal investigator	Group (if more than 1 Group listed in the byline) and/or Subgroup (eg, Steering Committee)
Marcelo	Losso		MD	Hospital General de Agudos JM Ramos Mejía CRS (AKA Hospital Ramos Mejía) (BA - Balvanera CRS)	Buenos Aires, ARG	Patient enrollment and data entering	COVID-19 Prevention Network
Isabel	Cassetti		MD	Helios Salud Dr. Stamboulian - Casa Central Location (BA - San Telmo CRS)	Buenos Aires, ARG	Patient enrollment and data entering	COVID-19 Prevention Network
Steven	Innes		MD	Emavundleni CRS	Cape Town, RSA	Patient enrollment and data entering	COVID-19 Prevention Network
Linda-Gail	Bekker		MD	Emavundleni CRS	Cape Town, RSA	Patient enrollment and data entering	COVID-19 Prevention Network
Sheetal	Kassim		MD	Groote Schuur HIV CRS	Cape Town, RSA	Patient enrollment and data entering	COVID-19 Prevention Network
Catherine	Orrel		MD	Groote Schuur HIV CRS	Cape Town, RSA	Patient enrollment and data entering	COVID-19 Prevention Network
Graeme	Meintjes		MD	Khayelitsha CRS / (CIDRI UCT)	Cape Town, RSA	Patient enrollment and data entering	COVID-19 Prevention Network
Amy	Ward		MD	Khayelitsha CRS / (CIDRI UCT)	Cape Town, RSA	Patient enrollment and data entering	COVID-19 Prevention Network
Andreas	Diacon		MD	TASK Central	Belville, Cape Town, RSA	Patient enrollment and data entering	COVID-19 Prevention Network
David	Wohl		MD	Chapel Hill CRS	Chapel Hill, NC, US	Patient enrollment and data entering	COVID-19 Prevention Network
Cindy	Gray		MD	Chapel Hill CRS	Chapel Hill, NC, US	Patient enrollment and data entering	COVID-19 Prevention Network
Chrinstine	Turley		MD	Atrium Health	Charlotte, NC, US	Patient enrollment and data entering	COVID-19 Prevention Network
Temitope	Oyedele		MD	AYAR at CORE CRS	Chicago, IL, US	Patient enrollment and data entering	COVID-19 Prevention Network
Babafemi O.	Taiwo		MD	Northwestern University CRS	Chicago, IL, US	Patient enrollment and data entering	COVID-19 Prevention Network
Karen	Krueger		MD	Northwestern University CRS	Chicago, IL, US	Patient enrollment and data entering	COVID-19 Prevention Network
Rick	Novak		MD	UIC Project WISH CRS	Chicago, IL, US	Patient enrollment and data entering	COVID-19 Prevention Network
Beverly E.	Sha		MD	Rush University CRS	Chicago, IL, US	Patient enrollment and data entering	COVID-19 Prevention Network

Supplemental Online Content: Nonauthor Collaborators
*First name, last name, and suffix (if applicable) are required and will appear in PubMed.

*First Name and Middle Initial(s)	*Last Name	*Suffix (eg, Jr, III)	Academic Degrees	Institution	Location (city, state/province, country)	Role or Contribution, eg, chair, principal investigator	Group (if more than 1 Group listed in the byline) and/or Subgroup (eg, Steering Committee)
Laura	Hammit		MD	Chinle Ctr. for American Indian Health	Chinle, AZ, US	Patient enrollment and data entering	COVID-19 Prevention Network
Carl	Fichtenbaum		MD	Cincinnati CRS	Cincinnati, OH, US	Patient enrollment and data entering	COVID-19 Prevention Network
David	Bernstein		MD	Cincinnati Children's Hospital Medical Center VTEU	Cincinnati, OH, US	Patient enrollment and data entering	COVID-19 Prevention Network
Jeffrey	Jacobson		MD	Case CRS	Cleveland, OH, US	Patient enrollment and data entering	COVID-19 Prevention Network
Dima	Dandachi		MD	University of Missouri/Columbia CRC- St. Louis University VTEU subsite	Columbia, MO, US	Patient enrollment and data entering	COVID-19 Prevention Network
Hasan	Naqvi		MD	University of Missouri/Columbia CRC- St. Louis University VTEU subsite	Columbia, MO, US	Patient enrollment and data entering	COVID-19 Prevention Network
Susan	Koletar		MD	Ohio State University CRS	Columbia, MO, US	Patient enrollment and data entering	COVID-19 Prevention Network
Nadine	Rouphael		MD	The Hope Clinic of the Emory Vaccine Center	Decatur, GA, US	Patient enrollment and data entering	COVID-19 Prevention Network
Sri	Edupuganti		MD	The Hope Clinic of the Emory Vaccine Center	Decatur, GA, US	Patient enrollment and data entering	COVID-19 Prevention Network
Thomas	Campbell		MD	University of Colorado Hospital CRS	Aurora, CO, US	Patient enrollment and data entering	COVID-19 Prevention Network
Elizabeth	Secord		MD	Wayne State University ATN CRS	Detroit, MI, US	Patient enrollment and data entering	COVID-19 Prevention Network
Samantha	Siva		MD	Botha's Hill CRS	Durban, RSA	Patient enrollment and data entering	COVID-19 Prevention Network
Nitesha	Jeenarain		MD	Chatsworth CRS	Durban, RSA	Patient enrollment and data entering	COVID-19 Prevention Network
Logashvari	Naidoo		MD	Chatsworth CRS	Durban, RSA	Patient enrollment and data entering	COVID-19 Prevention Network
Nigel	Garrett		MD	CAPRISA eThekwini CRS	Durban, RSA	Patient enrollment and data entering	COVID-19 Prevention Network
Nivashnee	Naicker		MD	CAPRISA eThekwini CRS	Durban, RSA	Patient enrollment and data entering	COVID-19 Prevention Network
Vimla	Naicker		MD	Tongaat CRS	Durban, RSA	Patient enrollment and data entering	COVID-19 Prevention Network

*First Name and Middle Initial(s)	*Last Name	*Suffix (eg, Jr, III)	Academic Degrees	Institution	Location (city, state/province, country)	Role or Contribution, eg, chair, principal investigator	Group (if more than 1 Group listed in the byline) and/or Subgroup (eg, Steering Committee)
Jayganthie	Naidoo		MD	Tongaat CRS	Durban, RSA	Patient enrollment and data entering	COVID-19 Prevention Network
Anamikah	Premrajh		MD	Tongaat CRS	Durban, RSA	Patient enrollment and data entering	COVID-19 Prevention Network
Emmanuel	Walter		MD	Duke Vaccine and Trials Unit CRS	Durham, NC, US	Patient enrollment and data entering	COVID-19 Prevention Network
Jeff	Henderson		MD	Missouri Breaks Ind Research Inc	Eagle Butte, SD, US	Patient enrollment and data entering	COVID-19 Prevention Network
Hugo	Tempelman		MD	Ndlovu Research Center / Elandsdoorn CRS	Elandsdoorn, RSA	Patient enrollment and data entering	COVID-19 Prevention Network
Abraham	Siika		MD	Moi University Clinical Research Centre	Eldoret, Kenya	Patient enrollment and data entering	COVID-19 Prevention Network
Evan	Anderson		MD	Emory Childrens Center - Vaccine Research Clinic	Atlanta, GA, US	Patient enrollment and data entering	COVID-19 Prevention Network
Brenda	Okech		MD	UVRI-IAVI HIV Vaccine Program LTD. CRS	Entebbe, Uganda	Patient enrollment and data entering	COVID-19 Prevention Network
Richard	Rupp		MD	Baylor VTEU Subsite: University of Texas Medical Branch (UTMB)	Galveston, TX, US	Patient enrollment and data entering	COVID-19 Prevention Network
Hana	El Sahly		MD	Baylor College of Medicine VTEU	Houston, TX, US	Patient enrollment and data entering	COVID-19 Prevention Network
Catherine M.	Healy		MD	Baylor College of Medicine VTEU	Houston, TX, US	Patient enrollment and data entering	COVID-19 Prevention Network
Robert	Arduino		MD	Houston AIDS Research Team CRS	Houston, TX, US	Patient enrollment and data entering	COVID-19 Prevention Network
Patricia	Winokur		MD	University of Iowa Vaccine Research and Education	Iowa City, IA, US	Patient enrollment and data entering	COVID-19 Prevention Network
Martin	Casapia		MD	ACSA CRS	Iquitos, Peru	Patient enrollment and data entering	COVID-19 Prevention Network
Gailen	Marshall		MD	University of Mississippi Medical Center	Jackson, MS, US	Patient enrollment and data entering	COVID-19 Prevention Network
Bhagyashri D.	Navalkele		MD	University of Mississippi Medical Center	Jackson, MS, US	Patient enrollment and data entering	COVID-19 Prevention Network
Sharla	Badel-Faeson		MD	Clinical HIV Research Unit (CHRU) / Helen Joseph CRS	Johannesburg, RSA	Patient enrollment and data entering	COVID-19 Prevention Network

*First name, last name, and suffix (if applicable) are required and will appear in PubMed.

***First Name and Middle Initial(s)**	***Last Name**	***Suffix (eg, Jr, III)**	Academic Degrees	Institution	Location (city, state/province, country)	Role or Contribution, eg, chair, principal investigator	Group (if more than 1 Group listed in the byline) and/or Subgroup (eg, Steering Committee)
Ian	Sanne		MD	Clinical HIV Research Unit (CHRU) / Helen Joseph CRS	Johannesburg, RSA	Patient enrollment and data entering	COVID-19 Prevention Network
Lee	Fairlie		MD	Wits RHI Shandukani Research Centre	Johannesburg, RSA	Patient enrollment and data entering	COVID-19 Prevention Network
Adeodata	Kekitiinwa		MD	Baylor-Uganda CRS	Kampala, Uganda	Patient enrollment and data entering	COVID-19 Prevention Network
Patricia	Ntege		MD	Baylor-Uganda CRS	Kampala, Uganda	Patient enrollment and data entering	COVID-19 Prevention Network
Peter J.	Elyanu		MD	Baylor-Uganda CRS	Kampala, Uganda	Patient enrollment and data entering	COVID-19 Prevention Network
Cissy K.	Mutuluuza		MD	Joint Clinical Research Centre	Kampala, Uganda	Patient enrollment and data entering	COVID-19 Prevention Network
Sandra	Rwambuya		MD	Joint Clinical Research Centre	Kampala, Uganda	Patient enrollment and data entering	COVID-19 Prevention Network
Philippa	Musoke		MD	MU-JHU Research Collaboration (MUJHU CARE LTD) CRS	Kampala, Uganda	Patient enrollment and data entering	COVID-19 Prevention Network
Deo	Wabwire		MD	MU-JHU Research Collaboration (MUJHU CARE LTD) CRS	Kampala, Uganda	Patient enrollment and data entering	COVID-19 Prevention Network
Barbara	Pahud		MD	Children's Mercy Hospital CRS	Kansas City, MO, US	Patient enrollment and data entering	COVID-19 Prevention Network
Mario	Castro		MD	Children's Mercy Hospital CRS; University of Kansas SOM	Kansas City, MO, US	Patient enrollment and data entering	COVID-19 Prevention Network
Fredrick	Sawe		MD	KEMRI/Walter Reed Project Research Center	Kericho, Kenya	Patient enrollment and data entering	COVID-19 Prevention Network
Samuel G.	Ouma		MD	KISUMU CRS	Kisumu, Kenya	Patient enrollment and data entering	COVID-19 Prevention Network
Taraz	Samandari		MD	KISUMU CRS	Kisumu, Kenya	Patient enrollment and data entering	COVID-19 Prevention Network
Grace	Mboya		MD	KISUMU CRS	Kisumu, Kenya	Patient enrollment and data entering	COVID-19 Prevention Network
Craig	Innes		MD	Aurum Institute Klerksdorp CRS	Klerksdorp, RSA	Patient enrollment and data entering	COVID-19 Prevention Network
Philip	Kotze		MD	Qhakaza Mbokodo Research Clinic CRS	Ladysmith, RSA	Patient enrollment and data entering	COVID-19 Prevention Network

*First name, last name, and suffix (if applicable) are required and will appear in PubMed.

***First Name and Middle Initial(s)**	***Last Name**	***Suffix (eg, Jr, III)**	Academic Degrees	Institution	Location (city, state/province, country)	Role or Contribution, eg, chair, principal investigator	Group (if more than 1 Group listed in the byline) and/or Subgroup (eg, Steering Committee)
Javier	Lama		MD	Barranco CRS	Lima, Peru	Patient enrollment and data entering	COVID-19 Prevention Network
Jorge	Sanchez		MD	CITBM – UNIDEC, Centro de Investigaciones Tecnológicas, Biomédicas y Medioambientales CRS	Lima, Peru	Patient enrollment and data entering	COVID-19 Prevention Network
Jorge	Gallardo		MD	CITBM – UNIDEC, Centro de Investigaciones Tecnológicas, Biomédicas y Medioambientales CRS	Lima, Peru	Patient enrollment and data entering	COVID-19 Prevention Network
Pedro	Gonzales		MD	San Miguel CRS	Lima, Peru	Patient enrollment and data entering	COVID-19 Prevention Network
Robinson	Cabello		MD	Via Libre CRS	Lima, Peru	Patient enrollment and data entering	COVID-19 Prevention Network
Raphael	Landovitz		MD	UCLA CARE Center CRS	Los Angeles, CA, US	Patient enrollment and data entering	COVID-19 Prevention Network
Michael P.	Dube		MD	University of Southern California CRS	Los Angeles, CA, US	Patient enrollment and data entering	COVID-19 Prevention Network
Tamela	Gould-Porter		MD	University of Southern California CRS	Los Angeles, CA, US	Patient enrollment and data entering	COVID-19 Prevention Network
Steve	Shoptaw		MD	UCLA Vine Street Clinic CRS	Los Angeles, CA, US	Patient enrollment and data entering	COVID-19 Prevention Network
Jesse	Clark		MD	UCLA Vine Street Clinic CRS	Los Angeles, CA, US	Patient enrollment and data entering	COVID-19 Prevention Network
Sheena	Kotze		MD	Synexus Stanza Clinical Research Centre CRS	Mamelodi, RSA	Patient enrollment and data entering	COVID-19 Prevention Network
Olivia	Kasselman		MD	Synexus Stanza Clinical Research Centre CRS	Mamelodi, RSA	Patient enrollment and data entering	COVID-19 Prevention Network
Katherine	Gill		MD	Masiphumelele CRS	Masiphumelele, RSA	Patient enrollment and data entering	COVID-19 Prevention Network
Maphoshane	Nchabeleng		MD	MeCRU CRS	Medunsa, RSA	Patient enrollment and data entering	COVID-19 Prevention Network
Aditya	Gaur		MD	St. Jude Children's Research Hospital CRS	Memphis, TN, US	Patient enrollment and data entering	COVID-19 Prevention Network
Carlos	Cabrera		MD	Unidad de Atención Médica e Investigación en Salud CRS	Merida, Mexico	Patient enrollment and data entering	COVID-19 Prevention Network
Susanne	Doblecki-Lewis		MD	UofMiami, IDRU at Jackson Mem Hospital CRS	Miami, FL, US	Patient enrollment and data entering	COVID-19 Prevention Network

Supplemental Online Content: Nonauthor Collaborators
*First name, last name, and suffix (if applicable) are required and will appear in PubMed.

*First Name and Middle Initial(s)	*Last Name	*Suffix (eg, Jr, III)	Academic Degrees	Institution	Location (city, state/province, country)	Role or Contribution, eg, chair, principal investigator	Group (if more than 1 Group listed in the byline) and/or Subgroup (eg, Steering Committee)
Susan	Kline		MD	University of Minnesota	Minneapolis, MN, US	Patient enrollment and data entering	COVID-19 Prevention Network
Scott	McClelland		MD	Ganjoni Clinic	Mombasa, Kenya	Patient enrollment and data entering	COVID-19 Prevention Network
Mandaliya	Kishorchandra		MD	Ganjoni Clinic	Mombasa, Kenya	Patient enrollment and data entering	COVID-19 Prevention Network
Pamela	Mda		MD	Nelson Mandela Academic Research Unit CRS	Mthatha, RSA	Patient enrollment and data entering	COVID-19 Prevention Network
Thozama	Dubula		MD	Nelson Mandela Academic Research Unit CRS	Mthatha, RSA	Patient enrollment and data entering	COVID-19 Prevention Network
Walter	Jaoko		MD	KAVI-Institute of Clinical Research, University of Nairobi	Nairobi, Kenya	Patient enrollment and data entering	COVID-19 Prevention Network
Spyros	Kalams		MD	Vanderbilt Vaccine CRS	Nashville, TN, US	Patient enrollment and data entering	COVID-19 Prevention Network
David	Haas		MD	Vanderbilt Vaccine CRS	Nashville, TN, US	Patient enrollment and data entering	COVID-19 Prevention Network
Vladimir	Berthaud		MD	Meharry [Clinical and Translational Research Center (CTRC), Meharry Medical College]	Nashville, TN, US	Patient enrollment and data entering	COVID-19 Prevention Network
Clarence	Creech		MD	Vanderbilt University Medical Center VTEU	Nashville, TN, US	Patient enrollment and data entering	COVID-19 Prevention Network
Sue Ellen	Abdalian		MD	New Orleans Adolescent Trials Unit CRS/Tulane University School of Medicine - Clinical Translational Unit (CTU)	New Orleans, LA, US	Patient enrollment and data entering	COVID-19 Prevention Network
Jessica	Justman		MD	Bronx Prevention Research Center CRS	New York, NY, US	Patient enrollment and data entering	COVID-19 Prevention Network
	Delafontaine		MD	New Orleans Adolescent Trials Unit CRS/Tulane University School of Medicine - Clinical Translational Unit (CTU)	New Orleans, LA, US	Patient enrollment and data entering	COVID-19 Prevention Network
Kristen	Marks		MD	Weill Cornell Chelsea CRS	New York, NY, US	Patient enrollment and data entering	COVID-19 Prevention Network
Sharon	Mannheimer		MD	Harlem Prevention Center CRS	New York, NY, US	Patient enrollment and data entering	COVID-19 Prevention Network
Hong	Van Tieu		MD	New York Blood Center CRS	New York, NY, US	Patient enrollment and data entering	COVID-19 Prevention Network

Supplemental Online Content: Nonauthor Collaborators
*First name, last name, and suffix (if applicable) are required and will appear in PubMed.

***First Name and Middle Initial(s)**	***Last Name**	***Suffix (eg, Jr, III)**	Academic Degrees	Institution	Location (city, state/province, country)	Role or Contribution, eg, chair, principal investigator	Group (if more than 1 Group listed in the byline) and/or Subgroup (eg, Steering Committee)
Magda	Sobieszczyk		MD	Columbia P&S CRS	New York, NY, US	Patient enrollment and data entering	COVID-19 Prevention Network
Mark	Mulligan		MD	Weill Cornell Uptown CRS	New York, NY, US	Patient enrollment and data entering	COVID-19 Prevention Network
Vanessa	Raabe		MD	NYU Manhattan Vaccine Center NYU VTEU	New York, NY, US	Patient enrollment and data entering	COVID-19 Prevention Network
Mary	Olson		MD	NYU Manhattan Vaccine Center NYU VTEU	New York, NY, US	Patient enrollment and data entering	COVID-19 Prevention Network
Juanita	Erb		MD	NYU Manhattan Vaccine Center NYU VTEU	New York, NY, US	Patient enrollment and data entering	COVID-19 Prevention Network
Shobha	Swaminathan		MD	New Jersey Medical School CRS	Newark, NJ, US	Patient enrollment and data entering	COVID-19 Prevention Network
Jose	Pilotto		MD	Hospital Geral de Nova Iguaçu CRS	Nova Iquaçu, Brazil	Patient enrollment and data entering	COVID-19 Prevention Network
Christopher	Hall		MD	East Bay AIDS Center (EBAC) CRS	Oakland, CA, US	Patient enrollment and data entering	COVID-19 Prevention Network
Diana	Florescu		MD	University of Nebraska	Omaha, NE, US	Patient enrollment and data entering	COVID-19 Prevention Network
Edwin	de Jesus		MD	Orlando Immunology Center CRS	Orlando, FL, US	Patient enrollment and data entering	COVID-19 Prevention Network
Ian	Frank		MD	Penn Prevention CRS	Philadelphia, PA, US	Patient enrollment and data entering	COVID-19 Prevention Network
Katie	Barr		MD	Penn Prevention CRS	Philadelphia, PA, US	Patient enrollment and data entering	COVID-19 Prevention Network
Deborah	McMahon		MD	University of Pittsburgh CRS	Pittsburgh, PA, US	Patient enrollment and data entering	COVID-19 Prevention Network
Sharon	Riddler		MD	University of Pittsburgh CRS	Pittsburgh, PA, US	Patient enrollment and data entering	COVID-19 Prevention Network
Judith M.	Martin		MD	Vanderbilt VTEU Subsite, University of Pittsburgh	Pittsburgh, PA, US	Patient enrollment and data entering	COVID-19 Prevention Network
Elizabeth	Barranco		MD	CAIMED-PHSU- SLU subsite	Ponce, PR, US	Patient enrollment and data entering	COVID-19 Prevention Network
Breno	Santos		MD	Hospital Nossa Senhora da Conceicao CRS	Porto Alegre, Brazil	Patient enrollment and data entering	COVID-19 Prevention Network

Supplemental Online Content: Nonauthor Collaborators
*First name, last name, and suffix (if applicable) are required and will appear in PubMed.

***First Name and Middle Initial(s)**	***Last Name**	***Suffix (eg, Jr, III)**	Academic Degrees	Institution	Location (city, state/province, country)	Role or Contribution, eg, chair, principal investigator	Group (if more than 1 Group listed in the byline) and/or Subgroup (eg, Steering Committee)
Karen	Tashima		MD	The Miriam Hospital CRS	Providene, RI, US	Patient enrollment and data entering	COVID-19 Prevention Network
Beatriz	Grinsztejn		MD	Instituto de Pesquisa Clinica Evandro Chagas (IPEC) CRS	Rio de Janeiro, Brazil	Patient enrollment and data entering	COVID-19 Prevention Network
Esau J.	Filho		MD	Hospital Federal dos Servidores do Estado NICHD CRS	Rio de Janeiro, Brazil	Patient enrollment and data entering	COVID-19 Prevention Network
Mike	Keefer		MD	University of Rochester Vaccines to Prevent HIV Infection CRS	Rochester, NY, US	Patient enrollment and data entering	COVID-19 Prevention Network
Ann	Falsey		MD	University of Rochester VTEU	Rochester, NY, US	Patient enrollment and data entering	COVID-19 Prevention Network
William	Brumskine		MD	Rustenburg CRS	Rustenburg, RSA	Patient enrollment and data entering	COVID-19 Prevention Network
Stuart	Cohen		MD	UC Davis MC	Sacramento, CA, US	Patient enrollment and data entering	COVID-19 Prevention Network
Jason	Okulicz		MD	San Antonio Brooke Army Medical Center	San Antonio, TX, US	Patient enrollment and data entering	COVID-19 Prevention Network
Barbara	Taylor		MD	UT Health San Antonio	San Antonio, TX, US	Patient enrollment and data entering	COVID-19 Prevention Network
Thomas	Patterson		MD	UT Health San Antonio	San Antonio, TX, US	Patient enrollment and data entering	COVID-19 Prevention Network
Ryan	Maves		MD	Naval Medical Center San Diego	San Diego, CA, US	Patient enrollment and data entering	COVID-19 Prevention Network
Susan	Little		MD	UCSD Antiviral Research Center CRS	San Diego, CA, US	Patient enrollment and data entering	COVID-19 Prevention Network
Susan	Buchbinder		MD	Bridge HIV CRS	San Francisco, CA, US	Patient enrollment and data entering	COVID-19 Prevention Network
Annie	Luetkemeyer		MD	UCSF HIV/AIDS CRS	San Francisco, CA, US	Patient enrollment and data entering	COVID-19 Prevention Network
Carmen	Zorrilla		MD	CEMI, MI-HMHR, IUPR-CTU, SJ ZIP Maternal-Infant Studies Center (CEMI)	San Juan, PR, US	Patient enrollment and data entering	COVID-19 Prevention Network
Esper	Kallas		MD	Centro de Pesquisas Clínicas IC-HCFMUSP CRS (Cerqueira Cesar)	Sao Paulo, Brazil	Patient enrollment and data entering	COVID-19 Prevention Network
Vivian A.	Silva		MD	Centro de Pesquisas Clínicas IC-HCFMUSP CRS (Cerqueira Cesar)	Sao Paulo, Brazil	Patient enrollment and data entering	COVID-19 Prevention Network

Supplemental Online Content: Nonauthor Collaborators
*First name, last name, and suffix (if applicable) are required and will appear in PubMed.

***First Name and Middle Initial(s)**	***Last Name**	***Suffix (eg, Jr, III)**	Academic Degrees	Institution	Location (city, state/province, country)	Role or Contribution, eg, chair, principal investigator	Group (if more than 1 Group listed in the byline) and/or Subgroup (eg, Steering Committee)
Tamara	Newman		MD	Instituto de Infectologia Emilio Ribas CRS	Sao Paulo, Brazil	Patient enrollment and data entering	COVID-19 Prevention Network
Jose	Valdez Madruga		MD	Centro de Referencia e Treinamento DST/AIDS CRS (Vila Mariana)	Sao Paulo, Brazil	Patient enrollment and data entering	COVID-19 Prevention Network
Julie	McElrath		MD	Seattle Vaccine and Prevention CRS	Seattle, WA, US	Patient enrollment and data entering	COVID-19 Prevention Network
Lisa	Jackson		MD	Kaiser Permanente Washington Health Research Institute VTEU	Seattle, WA, US	Patient enrollment and data entering	COVID-19 Prevention Network
Anna	Wald		MD	University of Washington VTEU	Seattle, WA, US	Patient enrollment and data entering	COVID-19 Prevention Network
Mookho	Malahleha		MD	Setshaba Research Centre CRS	Soshanguve, RSA	Patient enrollment and data entering	COVID-19 Prevention Network
Fatima	Laher		MD	Soweto HVTN CRS	Soweto-Bara, RSA	Patient enrollment and data entering	COVID-19 Prevention Network
Erica	Lazarus		MD	Kliptown Soweto CRS	Johannesburg, RSA	Patient enrollment and data entering	COVID-19 Prevention Network
Anusha	Nana		MD	Kliptown Soweto CRS	Johannesburg, RSA	Patient enrollment and data entering	COVID-19 Prevention Network
Daniel	Hoft		MD	St. Louis University VTEU	St. Louis, MO, US	Patient enrollment and data entering	COVID-19 Prevention Network
Sharon	Frey		MD	St. Louis University VTEU	St. Louis, MO, US	Patient enrollment and data entering	COVID-19 Prevention Network
Rachel	Presti		MD	Washington University Therapeutics (WT) CRS	St. Louis, MO, US	Patient enrollment and data entering	COVID-19 Prevention Network
Sharon	Nachman		MD	SUNY Stony Brook NICHD CRS	Stony Brook, NY, US	Patient enrollment and data entering	COVID-19 Prevention Network
Benjamin	Luft		MD	Stony Brook Responder Vaccine Program	Stony Brook, NY, US	Patient enrollment and data entering	COVID-19 Prevention Network
Carina	Rodriguez		MD	Univ. of South Florida (USF) College of Medicine ATN CRS	Tampa, FL, US	Patient enrollment and data entering	COVID-19 Prevention Network
Kathy	Mngadi		MD	The Aurum Institute Tembisa Clinical Research Centre Clinic 4 CRS	Tembisa, RSA	Patient enrollment and data entering	COVID-19 Prevention Network
Eric	Daar		MD	Harbor-UCLA CRS	Torrance, CA, US	Patient enrollment and data entering	COVID-19 Prevention Network

Supplemental Online Content: Nonauthor Collaborators
*First name, last name, and suffix (if applicable) are required and will appear in PubMed.

***First Name and Middle Initial(s)**	***Last Name**	***Suffix (eg, Jr, III)**	Academic Degrees	Institution	Location (city, state/province, country)	Role or Contribution, eg, chair, principal investigator	Group (if more than 1 Group listed in the byline) and/or Subgroup (eg, Steering Committee)
Shaun	Barnabas		MD	FAM-CRU (Family Clinical research Unit)	Tygerberg, RSA	Patient enrollment and data entering	COVID-19 Prevention Network
Mark	Cotton		MD	FAM-CRU (Family Clinical research Unit)	Tygerberg, RSA	Patient enrollment and data entering	COVID-19 Prevention Network
Stephen	Spector		MD	UCSD CRS Mother-Child Adolescent Program	San Diego, CA, US	Patient enrollment and data entering	COVID-19 Prevention Network
Karen	Servilla		MD	Raymond G Murphy VA MC	Albuquerque, NM, US	Patient enrollment and data entering	COVID-19 Prevention Network
Kathleen A.	Linder		MD	Ann Arbor VA Medical Center	Ann Arbor, MI, US	Patient enrollment and data entering	COVID-19 Prevention Network
Abeer	Moana		MD	Atlanta VA Medical Center	Atlanta, GA, US	Patient enrollment and data entering	COVID-19 Prevention Network
Carol	Kauffman		MD	Ann Arbor VA Medical Center	Ann Arbor, MI, US	Patient enrollment and data entering	COVID-19 Prevention Network
Mary	Bessesen		MD	Rocky Mountain Regional VAMC	Aurora, CO, US	Patient enrollment and data entering	COVID-19 Prevention Network
Rohit	Talwani		MD	VA Maryland Health Care System	Baltimore, MD, US	Patient enrollment and data entering	COVID-19 Prevention Network
Louis	Dell'Italia		MD	Birmingham VA Medical Center	Birmingham, VA, US	Patient enrollment and data entering	COVID-19 Prevention Network
Augusto	Alonto		MD	Jesse Brown VA MC	Chicago, IL, US	Patient enrollment and data entering	COVID-19 Prevention Network
Daniel	Liebman		MD	Columbia VA Health Care System	Columbia, SC, US	Patient enrollment and data entering	COVID-19 Prevention Network
Roger	Bedimo		MD	North Texas VA Health Care System	Dallas, TX, US	Patient enrollment and data entering	COVID-19 Prevention Network
Christopher	Woods		MD	Durham VA Medical Center	Durham, NC, US	Patient enrollment and data entering	COVID-19 Prevention Network
Peruvemba	Sriram		MD	North Florida South Georgia Veteran Health System	Gainesville, FL, US	Patient enrollment and data entering	COVID-19 Prevention Network
Kalpana	Padala		MD	Central Arkansas Veterans Healthcare System	Little Rock, AR, US	Patient enrollment and data entering	COVID-19 Prevention Network
Gregory	Holt		MD	Miami VA Medical Center	Miami, FL, US	Patient enrollment and data entering	COVID-19 Prevention Network

*First name, last name, and suffix (if applicable) are required and will appear in PubMed.

***First Name and Middle Initial(s)**	***Last Name**	***Suffix (eg, Jr, III)**	Academic Degrees	Institution	Location (city, state/province, country)	Role or Contribution, eg, chair, principal investigator	Group (if more than 1 Group listed in the byline) and/or Subgroup (eg, Steering Committee)
Edwin	Swialto		MD	Southeast Louisana VA Healthcare System	New Orleans, LA, US	Patient enrollment and data entering	COVID-19 Prevention Network
Melanie	Jay		MD	VA New York Harbor Healthcare System	New York, NY, US	Patient enrollment and data entering	COVID-19 Prevention Network
Sabrina	Felson		MD	VA New York Harbor Healthcare System	New York, NY, US	Patient enrollment and data entering	COVID-19 Prevention Network
Nora	Henderson		MD	VA New York Harbor Healthcare System	New York, NY, US	Patient enrollment and data entering	COVID-19 Prevention Network
Sheldon	Brown		MD	James J. Peters VA Medical Center	Bronx, NY, US	Patient enrollment and data entering	COVID-19 Prevention Network
Saumuel	Aguayo		MD	Phoenix VA Health Care System	Phoenix, AZ, US	Patient enrollment and data entering	COVID-19 Prevention Network
Mark	Riddle		MD	Reno VA Medical Center	Reno, NV, US	Patient enrollment and data entering	COVID-19 Prevention Network
Phyllis	Tien		MD	San Francisco VA Medical Center	San Francisco, CA, US	Patient enrollment and data entering	COVID-19 Prevention Network
John	Toney		MD	James A. Haley Veterans Hospital	Tampa, FL, US	Patient enrollment and data entering	COVID-19 Prevention Network
Halima	Dawood		MD	Vulindlela CRS	Vulindlela, RSA	Patient enrollment and data entering	COVID-19 Prevention Network
Disebo	Makhaza		MD	Vulindlela CRS	Vulindlela, RSA	Patient enrollment and data entering	COVID-19 Prevention Network
Hugh	Mighty		MD	Howard University School of Medicine	Washington, DC, US	Patient enrollment and data entering	COVID-19 Prevention Network
Siham	Mahgoub		MD	Howard University School of Medicine	Washington, DC, US	Patient enrollment and data entering	COVID-19 Prevention Network
Wes	Campbell		MD	Walter Reed National Military Medical Center	Washington, DC, US	Patient enrollment and data entering	COVID-19 Prevention Network
David	Diemert		MD	George Washington Univ. CRS	Washington, DC, US	Patient enrollment and data entering	COVID-19 Prevention Network
Tiffany	Schwasinger-Schmidt		MD	University of Kansas	Wichita, KS, US	Patient enrollment and data entering	COVID-19 Prevention Network
Caryn G.	Morse		MD	Wake Forest University Health Sciences	Winston-Salem, NC, US	Patient enrollment and data entering	COVID-19 Prevention Network

Supplemental Online Content: Nonauthor Collaborators
*First name, last name, and suffix (if applicable) are required and will appear in PubMed.

***First Name and Middle Initial(s)**	***Last Name**	***Suffix (eg, Jr, III)**	Academic Degrees	Institution	Location (city, state/province, country)	Role or Contribution, eg, chair, principal investigator	Group (if more than 1 Group listed in the byline) and/or Subgroup (eg, Steering Committee)
John W.	Sanders		MD	Wake Forest University Health Sciences	Winston-Salem, NC, US	Patient enrollment and data entering	COVID-19 Prevention Network
John	Williamson		MD	Wake Forest University Health Sciences	Winston-Salem, NC, US	Patient enrollment and data entering	COVID-19 Prevention Network
Angelique	Luabeya		MD	SATVI, Brewelskloof Hospital	Worcester, RSA	Patient enrollment and data entering	COVID-19 Prevention Network
Elizabeth	Bukusi		MD	KEMRI	Kisumu, Kenya	Patient enrollment and data entering	COVID-19 Prevention Network
Nelly	Mugo		MD	KEMRI/CCR Partners in Health R&D (PHRD)	Thika, Kenya	Patient enrollment and data entering	COVID-19 Prevention Network
Hannah	Kibuuka		MD	Makerere University Walter Reed Project	Kampala, Uganda	Patient enrollment and data entering	COVID-19 Prevention Network
Betty	Mwesigwe		MD	Makerere University Walter Reed Project	Kampala, Uganda	Patient enrollment and data entering	COVID-19 Prevention Network
Andrew	Kambugu		MD	Infectious Disease Institute (IDI)	Kampala, Uganda	Patient enrollment and data entering	COVID-19 Prevention Network
Apolo P.	Balyegisawa		MD	Infectious Disease Institute (IDI)	Kampala, Uganda	Patient enrollment and data entering	COVID-19 Prevention Network
Kathleen	Mullane		MD	University of Chicago	Chicago, IL, US	Patient enrollment and data entering	COVID-19 Prevention Network
Ben	Andagalu		MD	KEMRI/US Army Medical Research Directorate – Africa/Kenya - Kombewa Clinical Research Center	Kisumu, Kenya	Patient enrollment and data entering	COVID-19 Prevention Network
Lucas	Tina		MD	KEMRI/US Army Medical Research Directorate – Africa/Kenya - Kombewa Clinical Research Center	Kisumu, Kenya	Patient enrollment and data entering	COVID-19 Prevention Network
Nathaniel	Copeland		MD	KEMRI/US Army Medical Research Directorate – Africa/Kenya - Kombewa Clinical Research Center	Kisumu, Kenya	Patient enrollment and data entering	COVID-19 Prevention Network
Jack	Hutter		MD	KEMRI/US Army Medical Research Directorate – Africa/Kenya - Kombewa Clinical Research Center	Kisumu, Kenya	Patient enrollment and data entering	COVID-19 Prevention Network
Claudio L.	de las Casas		MD	Instituto de Investigacion Nutricional - Vanderbilt VTEU subsite	Lima, Peru	Patient enrollment and data entering	COVID-19 Prevention Network

Supplemental Online Content: Nonauthor Collaborators
*First name, last name, and suffix (if applicable) are required and will appear in PubMed.

***First Name and Middle Initial(s)**	***Last Name**	***Suffix (eg, Jr, III)**	Academic Degrees	Institution	Location (city, state/province, country)	Role or Contribution, eg, chair, principal investigator	Group (if more than 1 Group listed in the byline) and/or Subgroup (eg, Steering Committee)
Guillermo	Ruiz-Palacios		MD	Instituto Nacional de Ciencias Médicas y Nutrición -Emory VTEU subsite	Mexico City, Mexico	Patient enrollment and data entering	COVID-19 Prevention Network
Joseph J.	Eron		MD	Department of Medicine, University of North Carolina at Chapel Hill School of Medicine	Chapel Hill, NC, USA	Patient enrollment and data entering	COVID-19 Prevention Network
Judith S.	Currier		MD	Department of Medicine, David Geffen School of Medicine, University of California	Los Angeles, CA, USA	Patient enrollment and data entering	COVID-19 Prevention Network
Myron S.	Cohen		MD	Department of Medicine, University of North Carolina at Chapel Hill School of Medicine	Chapel Hill, NC, USA	Patient enrollment and data entering	COVID-19 Prevention Network
Kami	Kim		MD	Center for Global Health Infectious Disease Research, University of South Florida	Tampa, FL, US	Patient enrollment and data entering	COVID-19 Prevention Network
Richard N.	Greenberg		MD	University of Kentucky	Lexington, Kentucky, US	Patient enrollment and data entering	COVID-19 Prevention Network
Margaret	Kasaro		MD			Patient enrollment and data entering	COVID-19 Prevention Network
Sinead	Delaney-Moretlwe		MD			Patient enrollment and data entering	COVID-19 Prevention Network
Rodney	Dawson		MD			Patient enrollment and data entering	COVID-19 Prevention Network
Carmen	Paez		MD	CoVPN, VIDD, FHCC	Seattle, Washington, US	Study Coordination	COVID-19 Prevention Network
Nicole	Grunenberg		MD	CoVPN, VIDD, FHCC	Seattle, Washington, US	Study Coordination	COVID-19 Prevention Network
William O.	Hahn		MD	CoVPN, VIDD, FHCC	Seattle, Washington, US	Study Coordination	COVID-19 Prevention Network
Huub C.	Gelderbloom		MD	CoVPN, VIDD, FHCC	Seattle, Washington, US	Study Coordination	COVID-19 Prevention Network
Michele P.	Andrasik		PhD	CoVPN, VIDD, FHCC	Seattle, Washington, US	Study Coordination	COVID-19 Prevention Network
Stephaun E.	Wallace		PhD	CoVPN, VIDD, FHCC	Seattle, Washington, US	Study Coordination	COVID-19 Prevention Network
