# Supplementary material for: Rapid Development of an Integrated Network Infrastructure to Conduct Phase 3 COVID-19 Vaccine Trials
Source: JAMA Netw Open. Author manuscript; Available in PMC 2023 Oct 3. (PMC10546713; doi:10.1001/jamanetworkopen.2022.51974)
Supplement: Supplement 1 eMethods. Trial Protocols and Inception of the CoVPN — eMethods. Trial Protocols and Inception of the CoVPN [file NIHMS1927825-supplement-Supplement_1_eMethods__Trial_Protocols_and_Inception_of_the_CoVPN.pdf]

eMethods. Trial Protocols and Inception of the CoVPN

Moderna Protocol

https://www.nejm.org/doi/full/10.1056/nejmoa2035389#article_supplementary_material

Janssen Protocol

https://www.nejm.org/doi/full/10.1056/NEJMoa2101544#article_supplementary_material

Astrazeneca Protocol

https://www.nejm.org/doi/full/10.1056/NEJMoa2105290#article_supplementary_material

Novavax Protocol

https://www.nejm.org/doi/full/10.1056/NEJMoa2116185#article_supplementary_material

Sanofi Protocol

https://clinicaltrials.gov/ct2/show/NCT04904549

Expanding on the Inception of the CoVPN

Regulatory, pharmaceutical, and academic groups involved reviewed Phase 1/2 data in real time, ensuring rapid decision making in the selection of vaccine candidates from each manufacturer.

This inclusion of CRO-based clinical study sites permitted inclusion of clinical study sites beyond those affiliated with the CoVPN, allowing for faster and wider reaching enrollment than the CoVPN could achieve without these partnerships.

By bringing together these networks, the CoVPN was able to draw on existing infrastructure, community partnerships, and expertise, to quickly pivot clinical trial sites to conduct Covid-19 vaccine trials as soon as investigational products and protocols were ready for Phase 3 testing.

© 2023 Mena Lora AJ et al. *JAMA Network Open*.
