# Supplementary figures and images for: Rapid Development of an Integrated Network Infrastructure to Conduct Phase 3 COVID-19 Vaccine Trials
Source: JAMA Netw Open. Author manuscript; Available in PMC 2023 Oct 3. (PMC10546713; doi:10.1001/jamanetworkopen.2022.51974)

**eFigure.** Location of CoVPN Clinical Trial Sites Worldwide and in the US

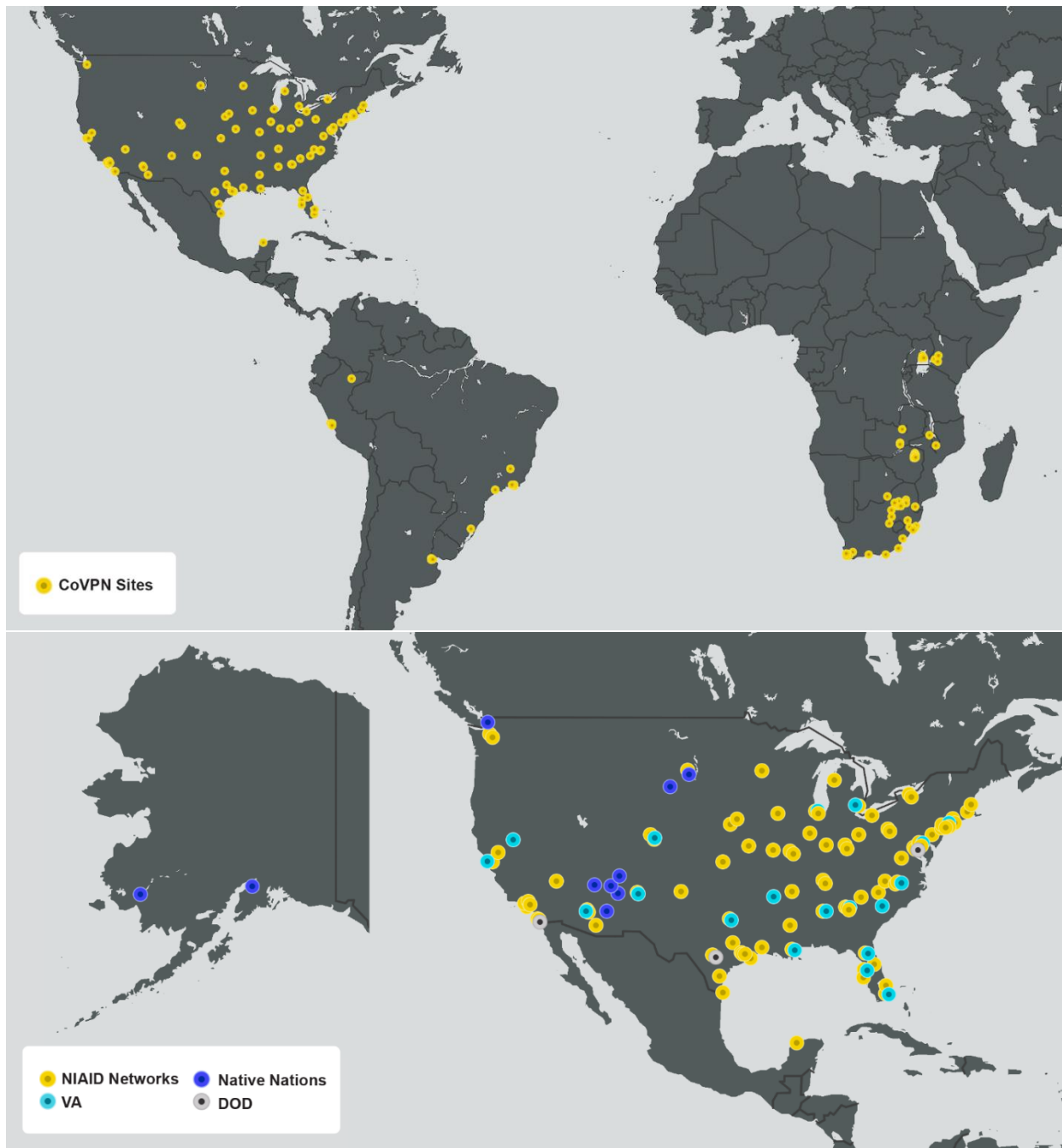

Supplement: Supplement 1 eFigure. Location of CoVPN Clinical Trial Sites Worldwide and in the US — eFigure. Location of CoVPN Clinical Trial Sites Worldwide and in the US [file NIHMS1927825-supplement-Supplement_1_eFigure__Location_of_CoVPN_Clinical_Trial_Sites_Worldwide_and_in_the_US.pdf]
