# Supplementary material for: Rapid Development of an Integrated Network Infrastructure to Conduct Phase 3 COVID-19 Vaccine Trials
Source: JAMA Netw Open. Author manuscript; Available in PMC 2023 Oct 3. (PMC10546713; doi:10.1001/jamanetworkopen.2022.51974)
Supplement: Supplement 1 eTable 1. Vaccine Platforms and Phase 3 Vaccine Efficacy Studies — eTable 1. Vaccine Platforms and Phase 3 Vaccine Efficacy Studies [file NIHMS1927825-supplement-Supplement_1_eTable_1__Vaccine_Platforms_and_Phase_3_Vaccine_Efficacy_Studies.pdf]

eTable 1. Vaccine Platforms and Phase 3 Vaccine Efficacy Studies

CoVPN Protocol	Company	Vaccine	Platform	Phase III Study
CoVPN 3001	Moderna	mRNA-1273	mRNA; lipid nanoparticle–encapsulated mRNA-based vaccine that encodes the pre-fusion stabilized full-length spike protein of SARS-CoV-2	COVE
CoVPN 3002	AstraZeneca/ Oxford	ChAdOx1 nCoV-19/AZD1222	Replication incompetent viral vector, Chimpanzee Adenovirus	AZD1222
CoVPN 3003	Janssen/J&J	Ad26.COV2.S	Replication incompetent viral vector, Adenovirus	ENSEMBLE
CoVPN 3004	Novavax	NVX-CoV2373	Recombinant protein with adjuvant; full-length, pre-fusion stabilized, recombinant spike protein. This is then assembled into nanoparticles with a saponin-based adjuvant (Matrix-M).	PREVENT-19
CoVPN 3005	Sanofi/GSK	CoV2 preS dTM-AS03	Recombinant protein with adjuvant; trimerized recombinant spike protein subunits co-formulated with AS03; . Stage 2 of the protocol included the assessment of the bivalent SARS-CoV-2 recombinant protein vaccine with AS03-adjuvant (5 µg of ancestral (D614) and 5 µg of B.1.351 [beta] variant spike protein)	VAT00008

© 2023 Mena Lora AJ et al. *JAMA Network Open*.
