# Supplementary material for: Rapid Development of an Integrated Network Infrastructure to Conduct Phase 3 COVID-19 Vaccine Trials
Source: JAMA Netw Open. Author manuscript; Available in PMC 2023 Oct 3. (PMC10546713; doi:10.1001/jamanetworkopen.2022.51974)
Supplement: Supplement 1 eTable 2. Demographic and Clinical Characteristics of Each Study Population at Baseline — eTable 2. Demographic and Clinical Characteristics of Each Study Population at Baseline [file NIHMS1927825-supplement-Supplement_1_eTable_2__Demographic_and_Clinical_Characteristics_of_Each_Study_Population_at_Baseline.pdf]

eTable 2. Demographic and Clinical Characteristics of Each Study Population at Baseline

As noted in the following table, the ethnic diversity in the US based COVE trial was high; with 20.5% of participants identifying as Latino or Hispanic, 10.2% Black and 4.6% Asian. The Astra Zeneca AZD1222 study, conducted in 88 sites in the US, Chile and Peru, enrolled 22.3% Hispanic and 8.3% Black, and 22.6% were ≥65 years old. With sites in Argentina, Brazil, Chile, Colombia, Mexico, Peru, South Africa, and the United States, the Janssen ENSEMBLE had 45.3% Hispanic or Latino subjects and 19.4% Black. Across all study sites, 19.5% of participants were ≥65 years old. The Novavax PREVENT-19 study had sites in the US and Mexico, enrolling 21.9% Hispanic and 11.8% Black participants. As PREVENT-19 began enrolling later than the previous studies, when emergency-use authorization (EUA) of other vaccines was already available for those ≥65 years old in the US, only 12.6% of the study population included this age group. The ongoing Sanofi study, VAT00008, has the largest geographical distribution of sites, enrolling at 98 sites across 11 countries in the Americas, Africa, Europe, and Asia. At the time of this report, enrollment demographic data were not yet available for VAT0008.

	**COVE**	**AZD1222**	**ENSEMBLE**	**PREVENT-19**
**Full analysis population[a]** -- no.	30,346	32,380	43,788	29,868
**Sex** – no. (%)				
Male	15,974 (52.6)	18,014 (55.6)	24,046 (54.9)	15,588 (52.2)
Female	14,372 (47.4)	14,366 (44.4)	19,735 (45.1)	14,280 (47.8)
Intersex	---	---	6 (0.0)	---
**Median age, yr** – no. (range)	52 (18 - 95)	51 (18 - 101)	40 (18 - 80)	47 (18 - 95)
**Age groups, yr** – no. (%)				
18-64	22,826 (75.2)	25,068 (77.4)	35,228 (80.5)	26,114 (87.4)
≥65	7,520 (24.8)	7,312 (22.6)	8,560 (19.5)	3,754 (12.6)
**Race** – no. (%)				
American Indian or Alaskan Native	234 (0.8)	1281 (4.0)	4143 (9.5)	1973 (6.6)
Asian	1,395 (4.6)	1,429 (4.4)	1,429 (3.3)	1,236 (4.1)
Black or African American	3,098 (10.2)	2,685 (8.3)	8,515 (19.4)	3,519 (11.8)
Multiracial	638 (2.1)	768 (2.4)	2455 (5.6)	482 (1.6)

© 2023 Mena Lora AJ et al. *JAMA Network Open*.

Native Hawaiian or Other Pacific Islander	68 (0.2)	81 (0.3)	103 (0.2)	70 (0.2)
Not reported/unknown	288 (0.9)	551 (1.7)	1442 (3.3)	176 (0.6)
Other	593 (2.0)	---	---	---
White	24,032 (79.2)	25,585 (79.0)	25,701 (58.7)	22,412 (75.0)
**Ethnicity** – no. (%)				
Hispanic or Latino	6,230 (20.5)	7,222 (22.3)	19,839 (45.3)	6,548 (21.9)
Not Hispanic or Latino	23,838 (78.6)	24,677 (76.2)	22,843 (52.2)	23,240 (77.8)
Not reported/unknown	278 (0.9)	481 (1.5)	1,106 (2.5)	80 (0.3)
**Country** – no. (%)				
United States	30,346 (100.0)	28,718 (88.7)	19,306 (44.1)	28,104 (94.1)
Brazil	---	---	7,279 (16.6)	---
South Africa	---	---	6,576 (15.0)	---
Colombia	---	---	4,248 (9.7)	---
Argentina	---	---	2,996 (6.8)	---
Peru	---	1,463 (4.5)	1,771 (4.0)	---
Chile	---	2,199 (6.8)	1,133 (2.6)	---
Mexico	---	---	479 (1.1)	1,764 (5.9)
**Baseline SARS-CoV-2 Status**[c] – (no. %)				
Negative	29,734 (98.0)	31,463 (97.2)	39,513 (90.2)	28,184 (94.4)
Positive	612 (2.0)	917 (2.8)	4,275 (9.8)	1,684 (5.6)

[a]Full analysis population: cohort of participants who had undergone randomization and received at least one dose of placebo or vaccine. These numbers are provided from final analyses and may differ from those in the primary analysis manuscripts for each manuscript.

[b]Race and ethnicity were classified according to NIH Notice Number: NOT-OD-15-089 and self-reported by study participants.

[c]Baseline negative SARS-CoV-2 status was defined differently by study. Typically, this baseline variable was defined using PCR testing as well as serology testing. Missing or not completed tests are categorized as negative.

© 2023 Mena Lora AJ et al. *JAMA Network Open*.
