# Supplementary material for: Rapid Development of an Integrated Network Infrastructure to Conduct Phase 3 COVID-19 Vaccine Trials
Source: JAMA Netw Open. Author manuscript; Available in PMC 2023 Oct 3. (PMC10546713; doi:10.1001/jamanetworkopen.2022.51974)
Supplement: Supplement 1 eTable 3. Clinical Trial Milestones — eTable 3. Clinical Trial Milestones [file NIHMS1927825-supplement-Supplement_1_eTable_3__Clinical_Trial_Milestones.pdf]

**eTable 3.** Clinical Trial Milestones

| Milestone | COVE | AZD1222 | ENSEMBLE | PREVENT-19 | VAT00008 |
|---|---|---|---|---|---|
| **Study start date** | July 27, 2020 | August 28, 2020 | September 21, 2020 | December 27, 2020 | May 26, 2021 |
| **Study pauses** | None | September 9, 2020: transverse myelitis | October 12, 2020: unexplained illness<br>April 13, 2021: blood clots | None | None |
| **Primary analysis[a] cutoff date** | November 25, 2020 | March 5, 2021 | January 22, 2021 | April 19, 2021 | [pending date] |
| **Final blinded phase efficacy data cutoff date** | March 26, 2021 | July 30, 2021 | July 9, 2021 | July 29, 2021 | In process |
| **EUA** | December 18, 2020 | None in US | February 27, 2021 | Application submitted | Application submitted |
| **Crossover[b] start date** | December 28, 2020 | None (EUA vaccines encouraged) | March 10, 2021 | April 20, 2021[c] | Planning is underway |
| **Booster shots** | September 23, 2021 | None (EUA vaccines encouraged) | October 1, 2021 | December 20, 2021[c] | Planning is underway |

[a]Primary analysis defined differently in each study.

[b]Blinded crossover was completed in the Novavax trial. For all other studies, "crossover" refers to vaccination offer to participants who received the placebo, but participants who originally received vaccination were not offered placebo. For ADZ122 participants, participants were unblinded ahead of schedule and prior to the primary analysis to allow receipt of EUA vaccines.

[c]Novavax dates are for the adult study; the pediatric expansion activities followed at a later date.

© 2023 Mena Lora AJ et al. *JAMA Network Open*.
