# Supplementary material for: Rapid Development of an Integrated Network Infrastructure to Conduct Phase 3 COVID-19 Vaccine Trials
Source: JAMA Netw Open. Author manuscript; Available in PMC 2023 Oct 3. (PMC10546713; doi:10.1001/jamanetworkopen.2022.51974)
Supplement: Supplement 1 eTable 4. Individual Study Site Enrollments by Phase 3 Clinical Trial [file NIHMS1927825-supplement-Supplement_1_eTable_4__Individual_Study_Site_Enrollments_by_Phase_3_Clinical_Trial.pdf]

eTable 4. Individual Study Site Enrollments by Phase 3 Clinical Trial

Moderna			
**USA**	**Site Name**	**Enrolled**	**CoVPN Site**
Anderson	Name Not Available	269	N
Atlanta	Children's Healthcare of Atlanta - Anderson, Evan	250	Y
	Grady Health System - Kelley, Colleen	152	Y
Aurora	University of Colorado Hospital - Campbell, Thomas	217	Y
Austin	Name Not Available	839	N
Baltimore	University of Maryland School of Medicine - Kotloff, Karen	443	Y
Banning	Name Not Available	303	N
Baton Rouge	Name Not Available	308	N
Binghamton	Name Not Available	477	N
Birmingham	Name Not Available	342	N
Boston	Brigham and Womens Hospital - Baden, Lindsey	632	Y
Cedar Park	Name Not Available	281	N
Chandler	Name Not Available	238	N
Chapel Hill	University of North Carolina at Chapel Hill - Gay, Cynthia	175	Y
Charlotte	Name Not Available	460	N
Chattanooga	Name Not Available	395	N
Chicago	Name Not Available	197	N
	UIC Project WISH CRS - Novak, Richard	296	Y
	University of Chicago - Mullane, Kathleen	126	Y
Cincinnati	Name Not Available	476	N
	Cincinnati CRS - Fichtenbaum, Carl	185	Y
Cleveland	Name Not Available	358	N
Colorado Springs	Name Not Available	269	N
Dallas	Name Not Available	447	N
DeLand	Name Not Available	647	N

© 2023 Mena Lora AJ et al. *JAMA Network Open*.

Decatur	Hope Clinic of The Emory Vaccine Center - Rouphael, Nadine	297	Y
Detroit	Name Not Available	710	N
Fayetteville	Name Not Available	362	N
Fort Worth	Name Not Available	230	N
Glendale	Name Not Available	262	N
Grand Island	Name Not Available	364	N
Greenville	Name Not Available	432	N
Hackensack	Name Not Available	153	N
Hollywood	Name Not Available	582	N
Houston	Name Not Available	413	N
	Baylor College of Medicine - El Sahly, Hana	271	Y
Jacksonville	Name Not Available	304	N
Knoxville	Name Not Available	248	N
La Jolla	University of California San Diego - Spector, Stephen	336	Y
La Mesa	Name Not Available	400	N
Laredo	Name Not Available	390	N
Las Vegas	Name Not Available	767	N
League City	University of Texas Medical Branch (UTMB) - Rupp, Richard	83	Y
Lenexa	Name Not Available	561	N
Little Rock	Name Not Available	400	N
Los Angeles	Name Not Available	60	N
	UCLA Vine Street Clinic - (CRS) - Clark, Jesse	194	Y
McAllen	Name Not Available	699	N
Medford	Name Not Available	422	N
Melbourne	Name Not Available	128	N
Metairie	Name Not Available	350	N
Miami	Name Not Available	456	N
	University of Miami - Doblecki-Lewis, Susanne	283	Y
Missouri	Name Not Available	321	N
Murray	Name Not Available	75	N

© 2023 Mena Lora AJ et al. *JAMA Network Open*.

Nashville	Vanderbilt University Medical Center - Creech, Clarence	294	Y
	Vanderbilt University Medical Center - Kalams, Spyros	182	Y
New York	Weill Cornell Chelsea - (CRS) - Marks, Kristen	50	Y
	Weill Cornell Medical College - Marks, Kristen	56	Y
Newark	New Jersey Medical School - Swaminathan, Shobha	57	Y
Newton	Name Not Available	310	N
Norfolk	Name Not Available	317	N
North Charleston	Name Not Available	403	N
Oklahoma City	Name Not Available	421	N
Omaha	Name Not Available	357	N
Orlando	Name Not Available	260	N
Petal	Name Not Available	173	N
Philadelphia	Penn Prevention - (CRS) - Frank, Ian	126	Y
Phoenix	Name Not Available	257	N
Pittsburgh	UPMC University Center - Martin, Judith	256	Y
Raleigh	Name Not Available	137	N
Redding	Name Not Available	270	N
Rockville	Name Not Available	686	N
Sacramento	Name Not Available	407	N
Saint Louis	Saint Louis University - Frey, Sharon	440	Y
Salt Lake City	Name Not Available	214	N
San Antonio	Name Not Available	424	N
San Diego	Name Not Available	456	N
Savannah	Name Not Available	293	N
Seattle	Kaiser Permanente - Seattle - Jackson, Lisa	188	Y
Sioux City	Name Not Available	300	N
Spartanburg	Name Not Available	464	N
Stockbridge	Name Not Available	401	N
Tomball	Name Not Available	411	N
Tucson	Name Not Available	912	N

© 2023 Mena Lora AJ et al. *JAMA Network Open*.

Washington	George Washington University - Diemert, David	349	Y
West Palm Beach	Name Not Available	436	N
Wichita	Name Not Available	247	N
Wilmington	Name Not Available	247	N
Winston-Salem	Name Not Available	287	N

Janssen		
**ARG**	**Enrolled**	**CoVPN Site**
Buenos Aires	325	Y
CABA	260	N
Capital Federal	227	N
Ciudad Autonoma Buenos Aires	1405	N
La Plata	394	N
Ramos Mejia	403	N
**BRA**	**Enrolled**	**CoVPN Site**
Barretos	93	N
Belo Horizonte	438	N
Belo Horizonte	400	Y
BrasÃlia	393	N
Campinas	387	N
CriciÃºma	141	N
CuiabÃ¡	122	N
Curitiba	396	N
Natal	103	N
Nova IquaÃ§u	199	Y
Porto Alegre	294	N
Porto Alegre	250	Y
Ribeirao Preto	351	N
Rio de Janeiro	1209	Y
Salvador	340	N
Sao Caetano do Sul	247	N
Sao Paulo	646	Y
SÃ£o JosÃ© do Rio Preto	380	N
SÃ£o Paulo	955	N
**CHL**	**Enrolled**	**CoVPN Site**
San Miguel	511	N
Santiago	266	N
Talca	268	N

© 2023 Mena Lora AJ et al. *JAMA Network Open*.

Temuco	95	N
**COL**	**Enrolled**	**CoVPN Site**
Barranquilla	799	N
Bogota	1360	N
Cali	617	N
Floridablanca	477	N
Medellin	788	N
Soledad	227	N
**MEX**	**Enrolled**	**CoVPN Site**
Cuernavaca	33	N
Merida	272	Y
Mexico	64	N
Monterrey	113	N
**PER**	**Enrolled**	**CoVPN Site**
Callao	76	N
Iquitos	490	Y
Lima	1226	Y
**USA**	**Enrolled**	**CoVPN Site**
Akron	55	N
Albuquerque	66	Y
Anaheim	494	N
Anderson	235	N
Ann Arbor	152	N
Atlanta	204	Y
Aurora	302	Y
Austin	136	N
Baltimore	71	Y
Binghamton	12	N
Birmingham	175	Y
Boston	309	N
Boston	205	Y
Bronx	56	Y
Charlotte	218	N
Chicago	791	Y
Cincinnati	908	N
Cleveland	220	N
Columbia	9	Y
Columbus	196	N
Corvallis	80	N
Dallas	96	N
Dallas	38	Y
Decatur	8	Y

© 2023 Mena Lora AJ et al. *JAMA Network Open*.

Deland	36	N
Detroit	390	N
Durham	59	Y
Fayetteville	254	N
Gainesville	134	Y
Grand Rapids	461	N
Hallandale Beach	368	N
Hollywood	281	N
Houston	305	N
Huntsville	166	N
Jackson	53	Y
Kansas City	49	N
Las Vegas	134	N
Lenexa	133	N
Lexington	893	N
Little Rock	10	Y
Long Beach	310	N
Louisville	60	N
Medford	113	N
Memphis	283	Y
Metairie	486	N
Miami	436	N
Minneapolis	236	N
Mount Pleasant	177	N
Murray	76	N
Nashville	92	Y
New Brunswick	839	N
New Orleans	405	Y
New York	128	Y
North Charleston	461	N
Omaha	1	N
Orlando	457	N
Orlando	71	Y
Peoria	32	N
Philadelphia	532	N
Philadelphia	160	Y
Phoenix	335	N
Phoenix	39	Y
Pinellas Park	232	N
Pittsburgh	172	Y
Portland	17	N
Raleigh	421	N

© 2023 Mena Lora AJ et al. *JAMA Network Open*.

Reno	39	Y
Rochester	509	N
Rockville	86	N
San Antonio	213	N
San Diego	326	N
San Diego	312	Y
San Francisco	101	Y
Seattle	73	Y
Spartanburg	183	N
St. Louis	477	Y
Stanford	206	N
Tampa	178	Y
The Villages	372	N
Tucson	401	N
Valparaiso	395	N
West Hollywood	247	N
West Jordan	68	N
Winston-Salem	20	Y
**ZAF**	**Enrolled**	**CoVPN Site**
Bellville, Cape Town	250	Y
Bloemfontein	458	Y
Cape Town	378	Y
Durban	237	Y
Elandsdoorn	614	Y
Johannesburg	867	Y
Klerksdorp	183	Y
Ladysmith	300	Y
Mamelodi	297	Y
Masiphumelele	248	Y
Medunsa	103	Y
Middelburg	661	N
Mthatha	61	Y
Port Elizabeth	118	N
Pretoria	363	N
Rustenburg	191	Y
Somerset West	358	N
Soshanguve	304	Y
Soweto-Bara	155	Y
Tembisa	303	Y
Tygerberg	131	Y
Vulindlela	110	Y
Worcester	55	Y

© 2023 Mena Lora AJ et al. *JAMA Network Open*.

AstraZeneca			
**CHL**	**Site Name**	**Enrolled**	**CoVPN Site**
Quillota	CENRESIN	522	N
Santiago	Facultad de Medicina Universidad de Chile	1190	N
	Hospital Luis Calvo Mackenna	494	N
**PER**	**Site Name**	**Enrolled**	**CoVPN Site**
Cercardo de Lima	Clinica Internacional	624	N
La Perla	Centro de Invetigaciones Medicas	237	N
San Isidro	Clinica Ricardo Palma	609	N
**USA**	**Site Name**	**Enrolled**	**CoVPN Site**
Name Not Available	AMR Coral Gables/Miami, Formerly Clinical Research of South Florida, an AMR company	268	N
Name Not Available	AMR East Wichita, Formerly Heartland Associates East Wichita, an AMR company	599	N
Name Not Available	AMR Knoxville, Formerly New Orleans Center for Clinical Research - Knoxville, an AMR company	644	N
Name Not Available	AMR Lexington, Formerly Central Kentucky Research Associates, an AMR company	220	N
Name Not Available	ActivMed Practices & Research, Inc. - PORTSMOUTH	1210	N
Name Not Available	Advanced Clinical Research	910	N
Name Not Available	Applied Research Center of Arkansas	219	N

© 2023 Mena Lora AJ et al. *JAMA Network Open*.

Name Not Available	Beaumont Health System	97	N
Name Not Available	Centex Studies Inc.	268	N
Name Not Available	Centex Studies, Inc.	318	N
Name Not Available	Clinical Research Associates, Inc.	401	N
Name Not Available	Clinical Research Partners, LLC	1801	N
Name Not Available	Clinical Trials of America LA, LLC	765	N
Name Not Available	Clinical Trials of South Carolina	694	N
Name Not Available	Cognitive Clinical Trials, LLC	1457	N
Name Not Available	East-West Medical Research Institute	44	N
Name Not Available	Hassman Research Institute	651	N
Name Not Available	HealthPartners Institute	787	N
Name Not Available	Hope Research Institute	236	N
Name Not Available	Indiana University	530	N
Name Not Available	JEM Research Institute	336	N
Name Not Available	Javara Inc	701	N
Name Not Available	MedPharmics LLC	132	N
Name Not Available	MedPharmics Ã¢â,¬â€œ Albuquerque	282	N
Name Not Available	Medical University of South Carolina (MUSC)	636	N
Name Not Available	Mercury Street Medical Group	361	N
Name Not Available	Omega Medical Research	366	N
Name Not Available	Oregon Health & Science University	111	N
Name Not Available	Pharmaron CPC, Inc.	25	N
Name Not Available	Spartanburg Medical Research	686	N
Name Not Available	Tekton Research	517	N
Name Not Available	Tekton Research, Inc	366	N
Name Not Available	The Iowa Clinic, PC	393	N

© 2023 Mena Lora AJ et al. *JAMA Network Open*.

Name Not Available	The University of Vermont Medical Center	285	N
Name Not Available	Tufts Medical Center, Inc. - PARENT	101	N
Name Not Available	University of Colorado at Denver and the Health Sciences	1060	N
Name Not Available	University of Michigan	214	N
Name Not Available	University of Wisconsin	347	N
Name Not Available	West Virginia Research Institute	393	N
Name Not Available	Western Connecticut Health Network	36	N
Baltimore	Johns Hopkins Bloomberg School of Public Health	371	Y
Boston	Fenway Community Health	211	Y
Chicago	AYAR at CORE CRS	257	Y
	Rush University Medical Center	160	Y
Cincinnati	Cincinnati Children's Hospital Medical Center	502	Y
Cleveland	Case Clinical Research Site	39	Y
Columbus	Ohio State University	135	Y
Dallas	Trinity Health and Wellness Center/AIDS Arms, Inc.	227	Y
Durham	Duke University Health System	149	Y
Houston	University of Texas-Houston Hariss County Psychiatric Center	126	Y
Kansas City	Kansas University-Wichita	82	Y
	University of Kansas Hospital	127	Y
	University of Kansas Medical Center Research Institute, Inc.	294	Y
Los Angeles	UCLA	139	Y

© 2023 Mena Lora AJ et al. *JAMA Network Open*.

	University of Southern California Medical Center	164	Y
New York	Bellevue Hospital	202	Y
	Bronx Prevention Research Center CRS	108	Y
	Columbia P&S CRS	260	Y
	Montefiore Medical Center	187	Y
	NYU Langone Brooklyn	200	Y
	NYU Langone Health Center	212	Y
	NYU Winthrop Hospital, Clinical Trails Center	131	Y
	New York Blood Center	231	Y
	VA NY Harbour Healthcare System	163	Y
Oakland	East Bay AIDS Center	110	Y
Orlando	Orlando Immunology Center	673	Y
Pittsburgh	University of Pittsburgh	200	Y
Rochester	Rochester General Hospital	120	Y
	University of Rochester Medical Center	693	Y
San Antonio	Joint Base San Antonio	245	Y
	Wilford Hall Medical Ctr	233	Y
San Diego	El Centro Regional Medical Center	183	Y
	Naval Medical Center San Diego	221	Y
	UCSD Antiviral Research Center (AVRC)	378	Y
San Francisco	Bridge HIV CRS	256	Y
	Zuckerberg San Francisco General Hospital	108	Y
Seattle	Seattle Vaccine and Prevention CRS	227	Y
Torrance	Lundquist Institute for Biomedical Innovation	366	Y

© 2023 Mena Lora AJ et al. *JAMA Network Open*.

	at Harbor-UCLA Medical Center		
Washington	Fort Belvoir Community Hospital	294	Y
	Walter Reed National Military Medical Center	324	Y

Novavax			
**MEX**	**Site Name**	**Enrolled**	**CoVPN Site**
Cuernavaca	Instituto Nacional de Salud Publica (INSP)	100	N
Guadalajara	PanAmerican Clinical Research Mexico S.A de C.V	257	N
Juriquilla	PanAmerican Clinical Research Mexico	247	N
Merida	Unidad de Atencion Medica e Investigacion en Salud (UNAMIS)	404	N
Mexico City	CAIMED Investigacion en Salud S.A de C.V	184	N
Veracruz	FAICIC S. DE R.L. DE C.V.	670	N
**USA**	**Site Name**	**Enrolled**	**CoVPN Site**
Akron	Synexus Clinical Research, US, Inc.	126	N
Anaheim	Anaheim Clinical Trials	318	N
Anderson	Synexus Clinical Research US, Inc.	79	N
Ann Arbor	VA Medical Center	134	Y
Atlanta	Atlanta Center for Medical Research	440	N
	Morehouse School Of Medicine	172	Y
	Ponce de Leon Center	250	Y
	Synexus Clinical Research US, Inc.	148	N
Aurora	University of Colorado Hospital CRS	288	Y
Austin	Benchmark Research	301	N
Baltimore	University Of Maryland School Of Medicine	515	Y

© 2023 Mena Lora AJ et al. *JAMA Network Open*.

Banning	Advanced Clinical Research	170	N
Baton Rouge	Meridian Clinical Research, LLC	103	N
Beachwood	Rapid Medical Research, Inc.	312	N
Boston	Beth Israel Deaconess Medical Center	114	Y
Bristol	PMG Research of Bristol	392	N
Chapel Hill	University of North Carolina	284	Y
Charleston	Medical University of South Carolina, SCTR Research Nexus	277	N
Charlotte	The Charlotte	289	Y
Chattanooga	WR Clinsearch, LLC	422	N
Cheney	MultiCare Institute for Research & Innovation	253	N
Chicago	Cedar Crosse Research Center	331	N
	Synexus	167	N
Chula Vista	GW Research Inc	289	N
Cincinnati	Sterling Research Group, Ltd	325	N
	Sterling Research Group, Ltd.	282	N
	Synexus Clinical Research US, Inc.	224	N
Colorado Springs	Lynn Institute of the Rockies	142	N
Columbia	The Curators of University of Missouri	103	Y
Columbus	IACT Health	414	N
DeLand	University Clinical Research	392	N
Detroit	Wayne State University/ Children's Hospital of Michigan	151	Y
Durham	M3	454	N
Eagle Butte	American Indian Clinical Trials Research Network	245	Y
Evansville	Synexus USA	183	N

© 2023 Mena Lora AJ et al. *JAMA Network Open*.

Fayetteville	Carolina Institute for Clinical Research	363	N
Fort Bragg	Womack Army Medical Center	262	N
Fort Worth	Benchmark Research	223	N
Gainesville	SIMED Health, LLC / SIMED Research	564	N
Gulfport	MedPharmics, LLC	114	N
Hallandale Beach	M D Clinical	446	N
Henderson	Synexus Clinical Research US, Inc.	328	N
Hollywood	Research Centers of America	375	N
Houston	Baylor College of Medicine	197	Y
	Texas Center for Drug Development, Inc	486	N
Iowa City	University of Iowa Medical Center	150	Y
Jacksonville	Jacksonville Center for Clinical Research	491	N
Knoxville	PMG Research, Inc. d/b/a PMG Research of Knoxville	387	N
La Mesa	Velocity Clinical Research	303	N
Lakeland	Meridien Research/Accel Research	177	N
Las Vegas	Clinical Research Consortium	63	N
Lenexa	Johnson County Clin	288	N
Little Rock	Lynn Institute of the Ozarks	140	N
	Preferred Research Partners, Inc.	131	N
Los Alamitos	WR	303	N
Los Angeles	National Research Institute	269	N
McAllen	Centex Studies, Inc	157	N
Medford	CRISOR, LLC	243	N
Memphis	Clinical Neurosciecne Solutions, Inc. dba CNS Healthcare	203	N

© 2023 Mena Lora AJ et al. *JAMA Network Open*.

Meridian	Advanced Clinical Research	341	N
Metairie	Med Pharmics, LLC	404	N
Mexico City	CAIMED Investigacion en Salud S.A de C.V	1	N
Miami	Miami Veterans Affairs Medical Center	143	Y
	Suncoast Research Associates, LLC	145	N
	Suncoast Research Group, LLC	567	N
Minneapolis	Synexus Clinical Research US, Inc.	130	N
	University of Minnesota	326	Y
Mokena	Providea Health Partners LLC	121	N
Nashville	Meharry Medical College	87	Y
New York	Weill Cornell Chelsea CRS	69	Y
Newport News	Health Research of Hampton Roads, Inc	320	N
Norfolk	Meridian Clinical Research	192	N
North Hollywood	Transitional Research Group, Inc.	251	N
Oklahoma City	Lynn Health Science Institute	307	N
	Medical Research International	338	N
Omaha	Meridian Clinical Research Associates, LLC	298	N
	University Of Nebraska Medical Center	505	Y
Orlando	Clinical Neuroscience Solutions Inc	256	N
	Headlands Research Orlando	123	N
Phoenix	HOPE Research Institute	71	N
Pinellas Park	Synexus Clinical Research US, Inc	124	N
Plano	Research Your Health	737	N

© 2023 Mena Lora AJ et al. *JAMA Network Open*.

Pomona	Empire Clinical Research	277	N
Ponce	Ponce School Of Medicine	294	Y
Providence	The Miriam Hospital	136	Y
Raleigh	M3 Wake Research, Inc	602	N
Rochester	Rochester Clinical Research, Inc.	398	N
Rocky Mount	PMG Research of Rocky Mount, LLC	219	N
Sacramento	Benchmark Research	123	N
	University of California Davis Medical Center	233	Y
San Antonio	Synexus, US	181	N
	University of Texas Health Science Center San Antonio	176	Y
San Diego	California Research Foundation	304	N
San Juan	Universidad de Puerto Rico	216	Y
Seattle	University of Washington VTEU	489	Y
Shreveport	Willis	103	N
Sioux City	Meridian Clinical Research	341	N
St. Louis	Sundance Clinical Research, LLC	122	N
Stockbridge	Clinical Research Atlanta	171	N
Stony Brook	Stony Brook WTC Health Program	396	Y
Tampa	Jedidiah Clinical Research	281	N
	University of South Florida	358	Y
Tempe	AMR Tempe Clinical Research Consortium	113	N
The Villages	Synexus Clinical Research US, Inc.	151	N
Tomball	DM Clinical Research	219	N
Valparaiso	Buynak Clinical Research, P.C.	237	N
Vestavia Hills	Accel Research Sites	120	N

© 2023 Mena Lora AJ et al. *JAMA Network Open*.

Vista	Synexus Clinical Research US, Inc.	106	N
Warwick	Velocity Clinical Research, Providence	432	N
Washington	Howard University Hospital Howard/ University College of Medicine	164	Y
West Jordan	Advanced Clinical Research	347	N
West Palm Beach	Comprehensive Clinical Trials, Llc	254	N
Wilmington	PMG Research of Wilmington, LLC	345	N
Woodland	Woodland Clinic Medical Group	210	N

Moderna			Janssen	
Countries	1		Countries	8
Cities	81		Cities	134
Sites	89		Sites	180
CoVPN Site	25		CoVPN Site	81
Enrolled	28,937		Enrolled	44,324
AstraZeneca			Novavax	
Countries	3		Countries	2
Cities	67		Cities	102
Sites	86		Sites	120
CoVPN Site	40		CoVPN Site	27
Enrolled	44,600		Enrolled	44,796

© 2023 Mena Lora AJ et al. *JAMA Network Open*.
